# Comparing International Models of Integrated Care: How Can We Learn Across Borders?

**DOI:** 10.5334/ijic.5413

**Published:** 2020-04-01

**Authors:** Carolyn Steele Gray, Nick Zonneveld, Mylaine Breton, Paul Wankah, James Shaw, Geoff M. Anderson, Walter P. Wodchis

**Affiliations:** 1 Bridgepoint Collaboratory for Research and Innovation, Lunenfeld-Tanenbaum Research Institute, Sinai Health System, US; 2 Institute of Health Policy, Management & Evaluation, University of Toronto, Toronto, CA; 3 Vilans, Centre of Excellence for Long-term Care, NL; 4 TIAS School for Business and Society/University of Tilburg, NL; 5 Université de Sherbrooke, CA; 6 Clinical Governance in Primary Health Care, CA; 7 Institute for Health System Solutions and Virtual Care, Women’s College Research Institute, Women’s College Hospital, CA; 8 Implementation and Evaluation Science, Institute for Better Health, Trillium Health Partners, CA

**Keywords:** scale and spread, integrated care, implementation, case study, international comparison

## Abstract

**Introduction:**

Providers, managers, health system leaders, and researchers could learn across countries implementing system-wide models of integrated care, but require accessible methods to do so. This study assesses if a common framework could describe and compare key components of international models of integrated care.

**Theory and methods:**

A framework developed for an international study of programs that address high needs high cost patients was used to describe and compare 11 case studies analyzed in two international research projects; the Implementing Integrated Care for Older Adults with Complex Health Needs (iCOACH) study in Canada and New Zealand, and the Vilans research group exploring models in the Netherlands. Comparative summaries were generated, with findings discussed at a 2019 International Conference on Integrated Care workshop.

**Results:**

The template was found to be useful to compare integrated case analyses in different contexts, and stands apart from other case comparison approaches as it is easily applied and can provide practical guidance for frontline staff and managers. Areas of improvement for the template are identified and two updated versions are presented.

**Conclusions and discussion:**

There is value to using a common template to provide guidance in international comparison of models of integrated care. We discuss the applicability of the approach to support scale and spread of integrated care internationally.

## Introduction

Health systems globally are still struggling to roll out system-wide models of integrated health and social care [1]. In part, this is attributable to a lack of understanding of what elements are important for successfully scaling up integrated health and social care initiatives [2,3], and how to overcome associated implementation challenges [4,5]. While examples of innovation exist, they often never expand beyond the pilot phase. Sharing knowledge across these examples may offer insights into how we can scale, spread and sustain innovations as a vital step towards broader health system transformation. This type of comparative work is represented in the approach taken by the World Health Organization (WHO) [6]. In the WHO practical guide to scaling up health service innovation, they suggest it is essential to have a clear idea of the core components of the innovation, the organization, and the environment (context) to inform the process of scaling up [6]. It is also useful to consider the needs of adopters and their role in adapting and spreading innovations [4].

Comparative case study approaches may offer promise in meeting these challenges by sharing successes and identifying causes of ineffective health reform efforts [7]. To unpack and understand the complexities of integrated models of care across different countries and jurisdictions, many studies have adopted comparative case approaches [8,9,10]. Comparative case study methods have a fairly long history and a robust methodology [11,12,13]. At their core, they seek to understand phenomena in context. As compared to other methodologies that may aim to control for the “noise” of external factors, case studies consider embracing the mess of context to be fundamental to our ability to understand not just what occurred, by why and under what circumstances [14,15]. Studying phenomena in context allows for collection of essential elements of innovation identified by the WHO to inform scale and spread.

Comparative analyses focusing on health system reform have evolved over the last 20 years beginning with a macro level policy focus. More recent studies have focused on meso-level organizational processes and practices [16]. Comparative case studies at the organizational level have been shown to provide valuable insights with regard to effectiveness of interventions in particular contexts, can contribute to theory building, and can be used to guide implementation of new models [17]. Numerous single and comparative case studies of integrated care have been conducted [18,19,20,21,22,23], and can facilitate learning across borders to build strong national knowledge [7]. However, the purpose of these approaches are often to evaluate programs (comparative case study methods) or to provide evidence to inform policy (comparative policy analysis) within a context, and are generally not intended to offer practical guidance to support scale and spread and to compare among different contexts. What is required are approaches to describe core components of the intervention, organizations and environments that can be applied by adopters, i.e. practitioners implementing on the ground.

This study marks an important step towards development of an international standard for reporting integrated care initiatives, building on tools and lessons learned in developing a template to describe how programs worldwide are addressing a common problem of more efficiently and effectively delivering integrated care to patients with high needs and high cost. Researchers at the University of Toronto in Canada developed a guide to create standardized descriptions of models across nation-states. These descriptions were intended to be easily accessible to providers and managers seeking to adopt models of integrated care in their own settings. This project was initially sponsored by the Commonwealth Fund in 2018. The present study aims to assess whether the same method could be applied to extract similar descriptions of integrated care cases that have been studied as part of unrelated large empirical comparative case studies. This work was driven by two research questions:

1. Can a standardized Case Template be used to describe models of care to extract comparable data from existing empirical case studies of integrated care?
  - What modifications and adaptations to the template may be required?
2. What are the recommendations for adopters, researchers and decision-makers who wish to use the Case Template?

### Describing models of integrated care to inform scale and spread

In Goodwin’s 2016 perspective paper regarding how we define and understand integrated care, he offers “at its simplest, integrated care is an approach to overcome care fragmentations” [24]. This “simple” statement is arrived at through an account of the multiple, complex ways health systems address fragmentation via different levels of integration (eg, micro vs. meso level), taking on different forms (eg, horizontal vs. vertical integration), and occurring at varying degrees of intensity. Different heuristic models and frameworks of integrated care are available to unpack this complexity, and help determine which factors should be understood when attempting to describe the salient features and activities of models of integrated care. However, if we are to use descriptions to inform scale and spread of models of care, we must look beyond simple descriptions of key features and better understand the dynamic.

Recent writing from Horton et al and the Health Foundation about the challenge of spreading complex programs such as integrated care has emphasized the difficulty in “codifying and replicating” complex interventions [4,25]. The difficulty in codifying interventions refers to the challenge of determining which features of the program are most relevant to describe, and the possibility that the features of a program that drive its success might not be those we expect. Horton et al. emphasize that in addition to the basic descriptive features of the design of a program, it is also important to outline the implementation processes or “social mechanisms” by which a program has worked [4]. Program descriptions must balance a tension between “loosening and tightening” the descriptions of an intervention in order to inform the effort to spread the intervention broadly. A “loosening” approach encourages local adopters to imagine transformations to the program that would promote its success locally, whereas a “tightening” approach emphasizes details about the exact implementation processes and relational contexts that made the program successful. If the conditions of initial programs can be fully implemented then the tightening approach is most useful, otherwise some extent of loosening is needed and the core activities that constitute the program must be described in a way that enables adopters to achieve specific related program goals with the resources available.

Keeping in mind these two essential factors, describing key elements of integrated care with attention to social mechanisms, the **Integrated Care Case Study Descriptive Template** was developed to enable comparison of integrated models of care across diverse geographies and contexts; describing 35 programs in 11 countries for the Commonwealth Fund.

## Developing of the Case Template

The initial development of the data collection template was completed by a team at the University of Toronto as part of a project funded by the Commonwealth Fund. This work built on an initial project with the University of Toronto and the Kings Fund, describing international cases of integrated care in Australia, Canada, the Netherlands, New Zealand, Sweden, The United Kingdom, and the United States [26]. In the Commonwealth Fund project, the team developed two separate templates; one for collecting data on design elements and activities of the program and another for collecting data on the policy context that supported the program. The construction of both of these data collection templates were based on literature reviews and expert opinion.

The design elements template drew heavily on the work of the Commonwealth Fund’s International Experts Working Group on Patients with Complex Needs report [27] and the survey was structured to assess 10 design dimensions that the report suggested were essential and grouped these into three broader areas: 1) population segmentation, 2) care coordination, and 3) patient and caregiver engagement.

The policy support template was focused on the external policy and incentives component of the consolidated framework for implementation research model [28] and was informed by the National Academy of Medicine report on integrated care [29]. The template identifies four policy categories: 1) finance and payment, 2) data infrastructure and data sharing, 3) workforce and 4) staffing, and governance and partnerships – and allows for identification and description of policies that were relevant to models of integrated care.

Table 1 summarizes the components of the Integrated Care Case Study Descriptive Template (for brevity, hereafter referred to as the Case Template).

**Table 1:** Integrated Care Case Study Descriptive Template.

Program Structure (design elements)	

*Segmentation*	Defining and applying rules to identify and recruit patients who are likely to benefit.
*Coordination*	A process for intake to characterize needs, mechanisms for coordination across institutions and sectors like health and social care.
*Patient and caregiver engagement*	Support for shared decision-making, self-management and support for caregivers.
*Measures*	How programs defined success, their level of maturity and any evaluation work conducted.
**Policy-related context**	

*Governance*	Governance structures in place to support the model of care. Could include committees and/or boards who meet regularly and review performance data.
*Data sharing*	Data and information sharing policies and processes in place related to patient care.
*Staffing*	Staffing needed to support the model of care, including strategies on how to organize and prepare staff.
*Financing*	Financing structures put in place to support the model of care. Includes attention to payment mechanisms, presence of well-defined budgets, and sustainability of funding.

While the components are separated here, it is recognized that they are also interrelated. For example, appropriate approaches to coordination and engagement are likely contingent on the types of patients and caregivers being served which is determined through the intake and recruitment process.

## Methods

### Approach

To answer our research questions, we applied the Integrated Care Case Study Descriptive Template to case studies conducted by the Implementing Integrated Care for Older Adults with Complex Health Needs (iCOACH) and Vilans research teams. Both these groups have conducted larger international case studies of integrated care undertaken with non-uniform and uniquely, locally defined approaches. We used the template to describe 9 integrated care cases from the iCOACH study which explore models in two jurisdictions in Canada (Ontario and Quebec, each with 3 cases) and in New Zealand (3 cases), as well as 2 cases from the Netherlands studied by the Vilans team.

### Setting: iCOACH and Vilans case studies

The iCOACH research team included researchers, decision-makers, trainees and patient and family representatives from Canada (Ontario and Quebec) and New Zealand to explore the implementation of integrated community-based primary health care for older adults with complex needs. The cornerstone work of the team has been in-depth case studies of 9 different integrated care models, 3 in each jurisdiction. The team took a whole systems approach to understand the cases, including patient and caregiver, provider, organizational, and system level factors that play a role in the implementation of the models of care. To meet project objectives a multi-method case study approach was used, collecting qualitative data (interviews), quantitative data (surveys), as well as document analysis from each case(29). Overviews of the methods, theoretical frameworks, cases, policy environments, and reflections from decision-makers, patients and caregivers can be found in the iCOACH special issue in IJIC [30].

The Vilans research team consists of researchers from the Netherlands, working on several national (diabetes networks [31], stroke services networks [32]) and international comparative case studies (SUSTAIN [20], ESN [33]) on the development and implementation of integrated care initiatives. The researchers use a comprehensive multi-method case study approach. Both quantitative (surveys) and qualitative data (interviews, field notes) were collected from multiple perspectives (service users, professionals, managers as well as decision-makers).

All 9 of the iCOACH cases, and 2 cases from the Netherlands were included in the analysis presented here. While these cases were purposefully selected to answer the original research questions (see aforementioned publications), for the purposes of the case comparison study presented in this paper, case selection is more aligned with a convenience and purposeful approach [14] as they had sufficient data readily available to complete the Case Templates. Additionally, these were cases highly familiar to the study team as they had each engaged in setting up the original studies, collecting data and/or analyzing data for other studies. This afforded the team a wealth of context knowledge around the cases required to align available data to the template.

### Data extraction and analysis

The Case Template was originally created as a structured interview guide conducted in two parts. Key informants with knowledge of the models of care would start by rating their models along the four components in each section, and then would be asked probing question to elicit greater detail. See Supplementary Material 1 for an overview of the original interview guide questions and probes. We adapted this method, using the guide to “interview” ourselves, using the data collected in our case studies to answer questions and probes.

For the present study, research leads with in-depth knowledge of the cases in each jurisdiction (Ontario, Quebec, New Zealand or Netherlands) were assigned to complete Case Templates for cases in their area of expertise. While we did not have one of the local New Zealand research team members available to participate in this work, the Ontario team members participating in this study had previously conducted much of the initial coding and analysis of New Zealand data and had been working closely with New Zealand research team members, providing them the necessary knowledge and expertise of the models to conduct this work. Leads looked at case study data collected as part of the iCOACH project and similar data from Vilans integrated care case studies. Various data sources were reviewed to complete templates for each jurisdiction, including: published articles based on the case studies, documents collected as part of case study work (eg, vision and mission statements, relevant policy documents and websites), coded interview data from interviews with providers and managers, and, where required to fill gaps, original interview transcripts were reviewed. Research leads used these data sources to write answers directly into the structured interview guide for each of the 11 cases reviewed, and maintained analysis memos to track data sources, the process taken, and preliminary analytic thoughts.

To begin a single Case Template was completed for each of the four jurisdictions and circulated to the team for discussion regarding process and preliminary analytic reflections. Once we were satisfied that a similar process was being used across jurisdictions, the remaining cases were completed following the same procedure. With all templates completed the lead author distilled data into a single table to facilitate cross case comparison. The table was circulated to the team for review, followed by an analysis meeting where similarities and differences across the cases were discussed and agreed upon.

### Expert discussion and review

The above process and key results were presented in a 90 minute workshop at the International Conference on Integrated Care held in San Sebastian, Spain April 1^st^ 2019. In the workshop delegates were presented with the framework, an overview of the cases, our methods for comparing cases, and key results (presented in the results section). Delegates attending the workshop included researchers working in the field of integrated care, policy-makers and other decision-makers, as well as managers and front-line providers/practitioners engaged in delivering integrated care in their respective countries, representing all corners of the world including Europe, North America, Australia/Oceania and South-East Asia. Workshop participants had an opportunity to apply the Case Template to their own cases and engaged in roundtable discussions to help us address research questions regarding adaptation and recommendations for using the template. Workshop facilitators took notes at the session, and co-authors engaged in a post-workshop discussion to identify key learning from the exercise. While no formal ethics process was followed as the conversation was not recorded and names were not collected, delegates were made aware that the discussion would inform the refinement of the Case Template and be included as part of the publication.

## Results

The Case Template provided a useful lens to explore the 11 international cases. Table 2 offers a high level summary of the data across cases, with a full dataset available in Appendix A. The full data set was used to generate analytic discussion across the team. The following two sections highlight key findings from parts 1 and 2 of the Case Template and demonstrate its ability to be used to describe case studies in a comparable way. The 11 case studies represent different models of integrated care; for simplicity we refer to the case examples as “models” of care or cases.

**Table 2:** Sample case descriptions [for full descriptions see Appendix A].

Case	Segmentation	Coordination	Engagement Success	measures	Policy Context

SUSTAIN						

South Holland	***Target group:*** frail, multiple health and social care needs (but broadly defined) ***Entry points:*** self-referral (clients and families) or by professionals in the community (prevention driven with active community communication)	***Intake:*** Conducted by any provider using a standard tool (Self-reliance matrix) filled out during a home visit (using a tablet). Patient then assigned navigator (anyone on the team, all trained for this role – likely with most experience with care needs of patient). ***Primary care providers:*** Each team includes community nurse or GP practice nurse. Direct connection to GP practice varies. ***Integration:*** At minimum social worker, community nurse and municipal social care worker. Can add: dementia case managers, physicians, social housing, etc… ***Transitions:*** All providers still linked to their parent organizations which can facilitate transition ***Information sharing:*** All providers can access a shared data platform which includes online communication tool (all teams trained on it).	***Patient engagement:*** A strong belief of the teams, but not formalized. Also engagement is limited due to the low functional status of clients. ***Patient self-management:*** Similar to issue above, believed to be important but difficult to operationalize. Additional challenge of different characteristics of neighbourhoods. ***Caregiver engagement:*** Not yet a clear component of strategy – but family issues captured as part of the assessment process. Experimenting with digital tools to support caregiver engagement (interest in building this long term).	***Maturity:*** Program admitted first client in 2015 and has served 5,000 since. Served approx. 300 in the past 6 months. Program started as a pilot (3 teams) and scaled up in 2016 (27 teams). Composition, objectives and aims of teams varies by neighbourhood. ***Measures:*** Better health outcomes, patient/caregiver experience and lower costs – these are not formalized in measures (not unusual). ***Data collection:*** No data on program activities are collected. Currently developing performance indicators. ***Evaluation:*** No formal evaluation conducted.	***Financing for model:*** Both municipal (public tender and subsidized funds) and health insurer financed. ***Staffing model:*** All professionals stay employed by their mother organizations. Next to their daily work, they get extra hours for doing the multidisciplinary work/meetings. Professional training is executed by the local (applied) university and funded by the municipality. ***Governance structure:*** Shared governance model. All involved parties (health and social care providers, GPs, municipality, health insurers) are represented in a steering group. However, the two financing institutes (insurers and local government) are directing. No performance data is collected yet. ***Health and social care data sharing structure:*** To facilitate data linkage, a shared IT system has been developed. However, ‘old’ systems are still being used. Administrative burden is a risk. ***Care delivery innovation:*** Most innovative part is that a person/family has 1 contact person, and that integration takes place in all phases of the process: from intake to care delivery.
**ONTARIO**					

Integrated Client Care Program (ICCP) – partnership model between primary care and home care	***Target group:*** The Integrated Client Care Program (ICCP) focuses on the top 1–5% frail older adults in need of integrated services. Patients are assessed up the RAI tool. ***Entry points:*** Patients can enter the program through multiple entry points including the Family Health Team (FHT), the Community Care Access Centre (CCAC - government agency that connects patients to home care services), and through other partners and community agencies aware of the program.	***Intake:*** Intake depends on the RAI evaluation (see target group). The Care Coordinator from the CCAC typically takes responsibility for ICCP patients. ***Primary care providers:*** All patients on the ICCP program have sustained access to their primary care provider who is supported through a multi-disciplinary team ***Integration:*** There is a high level of professional integration with the multi-disciplinary team, as well as organizational integration between the FHT, the CCAC and hospital. ***Transitions:*** There is a formal program with the local hospital, Virtual Ward, which supports transitions for clients going from hospital to home. This is a clear process and protocol but only for patients at the local hospital – if patients end up in another hospital there is no process. ***Information sharing:*** Partnering organizations have connecting information systems (hospital and FHT), or individuals able to access multiple platforms (embedded care coordinator can see FHT and CRIS systems)	***Patient engagement:*** Patients engagement occurs at this site and is an increasing focus. There are patient and family carer seats on committees and strategic planning groups. ***Patient self-management:*** Collaboration and patient goal-setting is a part of the culture at the FHT and embedded into the ICCP program. ***Caregiver engagement:*** Caregiver support less formalized, but providers are attentive to caregiver needs and attempt to provide supports when they can. Not a formal process.	***Maturity:*** The ICCP program began in 2012 and is a replication from the ICCP program in palliative care run out of the CCAC. Other FHT programs like Virtual Ward and IMPACT are also established and support the integrated model. IMPACT is a replication from another site. ***Measures:*** Standard FHT measures apply to the FHT for reporting to the LHIN on performance. It is noted by decision-makers that other measures are currently missing, but they would anticipate that reduced hospitalizations and ER visits be among their key measures. ***Data collection:*** Data not available ***Evaluation:*** ICCP was not formally evaluated at the time of data collection. A different Virtual Ward program at Women’s College has had a formal evaluation, as has the IMPACT program in other settings.	***Financing for model:*** The ICCP program is funded by the FHT and CCAC through paying for specific staff to run the program. For the FHT the staff is now part of the global budget. ***Staffing model:*** Unique staffing model which collocates the community partner (home care coordinator) in the multi-disciplinary primary care team to improve coordination and information sharing. ***Governance structure:*** The FHT, like other FHTs in Ontario has a board of directors that reviews performance metrics aligned with Ministry reporting requirements. ***Health and social care data sharing structure:*** Some innovative data sharing between the FHT and the local hospital (sharing medical records), electronic referral and information sharing with Toronto EMS (paramedics), and colocation of staff enables seeing health and social care data while in the primary care clinic. ***Care delivery innovation:*** The colocation model of ICCP, along with the virtual care and home visiting programs are innovative practices in Ontario.
**QUEBEC**					

	***Target group:*** Functional Autonomy Measuring System (SMAF) used to determine eligibility – need a particular score to be included.	***Intake:*** SMAF scores guides a multidisciplinary care plan. Host organization and local organizations may have some flexibility in what is provided. Those with a SMAF >5 receive a case manager through home care services unit.	***Patient engagement:*** Personalized care plan but shared-decision making difficult to operationalize. Culture of shared decision-making supported by government and leaders.	***Maturity:*** CLSC’s operational since 1970s with 100,000’s since than. It is an established government run program with secure funding and spread across the province.	***Financing for model:*** Public fund troughs taxation. In complementary, patient may directly pay for services from community agencies that are mostly not covered by the public insurance.
	***Entry points:*** Patients with 2 or more YES answers on PRIMSA-7. SMAF is managed by a specialized team at a single point of entry for defined geography. Clients can also self-refer	***Primary care providers:*** Some regular contact but challenging to connect to primary care as they are privately owned. Case managers have primary responsibility.	***Patient self-management:*** No clear self-management support aspects of program	***Measures:*** Better health outcomes, patient and caregiver experience and lower costs. Related to government healthy aging policy with specific indicators: reduced wait times, reduced ED visits, # clients in the program.	***Staffing model:*** All professionals stay employed by their mother organization. Recent initiatives are in place to “lend” allied professionals (nurses, social workers, dieticians etc.) to privately owned Grouped Medical practices – the allied professional are still employed by the mother organization but work in private physician clinics. Family physicians in the community are paid through public insurance but are autonomous workers.
		***Integration:*** Types of services offered varies by local organization but all include primary care in the community, acute and surgical, home care, nursing home, supportive housing, community day care and social supports. Some co-location but not in all sites. ***Transitions:*** Some organizations have dedicated care transitions provider (engage in pre-discharge meetings) ***Information sharing:*** Have ICT systems to facilitate integration and transitions, in particularly tools that send transfer information electronically. Government mandated (RSIPA system). Some variation in access due to location.	***Caregiver engagement:*** Some caregiver supports offered (e.g. respite days) – no information regarding formal training.	***Data collection:*** Performance indicators reported on regularly. ***Evaluation:*** Several formal research studies conducted to evaluate the model. Developed OSIRSIPA tool to monitor implementation and outcomes.	***Governance structure:** Since 2015*, Almost a full integration of public establishment under the same governance (hospital, rehabilitation, home care, long care term facilities) Vertical governance structure. The HSSCs are public health and social care agencies that are mandated by the government to organize care delivery in their territories. The HSSCs have to lead in establishing local joint governance boards for various health problems with their local partners in the community (physician clinics, nursing homes, private community agencies etc.). ***Health and social care data sharing structure:*** There is a government mandated IT system (the RSIPA) that is shared between various agencies within the HSSC. However, some private agencies do not have access to this public IT system. Furthermore “older” IT systems co-exist with the public IT system. ***Care delivery innovation:*** Introduction of several initiatives. E.g. formalization of care coordination by case managers, use of multidisciplinary individualized service plans, and use of multidisciplinary health and social care evaluation tools.
**NEW ZEALAND**					

NZ Network Model	***Target group:*** The DHB serves a broad population but the CREST and care coordination programs focus on 65 and older population transitioning home from hospital. ***Entry points:*** Clients access services through Liaison Nurse who identifies eligible individuals in the hospital. Referrals for case management and care coordination programs for older adults can come through GPs, other providers or through self-referral.	***Intake:*** Assessments used by Liaison nurses and care coordinators to assign services based on function and need (eg, interRAI) ***Primary care providers:*** GPs play an active role in NZ Network Model, referring patients as needed to programs and following up with other providers. They will engage in case conference calls with other providers as well. ***Integration:*** Involves a wide range of health as social care services some of which are tailored to older adults with complex care needs. Providers regularly speak across boundaries to deliver care. ***Transitions:*** CREST is a structured transition program from hospital to home. Care coordinators and case managers work to help integrate other services. Teams across services also work together. ***Information sharing:*** Use a few systems to share information include CCMS, SAP, Momentum, Health Conenct South and One Health Now. Providers can access patient data that sits on these systems from different settings (eg, pharma, labs, clinical care, hospitals).	***Patient engagement:*** Goal-setting part of care delivery (particularly for CREST programs), not part of DHB training but embedded in professional training and approach. ***Patient self-management:*** Area of focus particularly for the CREST program with an emphasis on enablement and support. ***Caregiver engagement:*** Not an emphasis	***Maturity:*** New model in DHB established in 2006/7 but gained traction in 2011 post earthquakes. An established program with ongoing funding. ***Measures:*** Emphasis on process measures (early discharge), also collect patient satisfaction and engage in peer review meetings ***Data collection:*** No regular reporting mentioned in interviews – but likely occurring particularly for funded partners ***Evaluation:*** No formal evaluation to our knowledge	***Financing for model:*** DHB shifted to activity-based payment model for hospitals and bottom-up focused alliance contracting where maximum collective gain can only be realised if all parties support one another and agree to share any losses ***Staffing model:*** Unchanged – what has changed is how they work together ***Governance structure:*** Shift towards a Network model reliant on partnerships and governed by Alliance Support team. ***Health and social care data sharing structure:*** Not necessarily new but part of the NZ approach to data where patients have unique identifiers across health and social care data platforms to facilitate finding information. ***Care delivery innovation:*** Most notable shift is in moving clients out of hospital and into the community setting faster through partnerships with social care providers and enablement program (CREST)

### Comparing model structure features: Segmentation, Coordination, Engagement, Measurement

The models reflected in case studies followed various segmentation approaches. While all models cover a specific geographical area, they differ in their target group focus. In some cases, models have a broad scope, serving local communities as a whole (Community Health Centre, the Maori health organization); whereas other models focus on a more specific population. For instance, the CREST and care coordination programs in the NZ Network model, focus on people of 65 and older transitioning home from hospital. There were also examples of, “in between” models that focus on frail people or older people as wider target groups. When looking at the entry of these people into models, three categories can be distinguished: 1) professional entry, 2) self-entry, and 3) a combination of both. The Integrated Client Care Program (ICCP) model in Ontario, for example, can only be accessed through professional entry points. In the Community Health Centre model, on the other hand, both self-entry and professional entry are possible, but access is subject to availability and wait-lists. In other programs, such as South Holland, Quebec and the Maori health organization, both professional and self-entry are used.

*“People or their family and friends can refer themselves to the program by visiting the municipal single access point (visit, phone and online). People can also be referred by professionals working in their neighborhood, having an active signaling/preventing role.”* [South Holland program].

After entering the program, intake processes take place in all cases. Our analysis shows a broad spectrum of formal and less formal ways to conduct an intake. Some initiatives established standardized processes using validated instruments, such as the Functional Autonomy Measuring System (SMAF) guiding the development of multidisciplinary care plans in Quebec. The use of this clinical tool to assess the level of autonomy of older adults was mandated to all programs in Quebec. In other models, such as in the Community agency lead model in Ontario, the intake is an informal process and varies from program to program.

Although some variations in consistency and access are reported, in 10 out of 11 cases information sharing takes place through shared or linked digital data platforms to some degree. Only in the Utrecht Hills case is it described that professionals are not allowed to electronically share information and therefore rely on multi-disciplinary meetings occurring every six weeks.

Although many programs state that their practice is strongly driven by a belief in patient engagement, self-management and caregiver engagement, most report that few formal activities to achieve this have been implemented. Some models stress that goal-setting with patients is part of the working processes and happens regularly (e.g. the Maori health organization, ICCP). Other models report educational materials for patients (Community agency lead model, Ontario), information, advice, guidance and support for caregivers (Utrecht Hills) and respite programs for caregivers (Quebec programs). One program, ICCP, has organized patient and family caregiver roles on committees and strategic planning groups.

*“Government emphasizes shared decision-making, which is martialized by the personalized care plan. The operationalization of a “shared decision-making” concept is often difficult. Influenced by provider’s time pressure, case-loads, characteristics of clients (cognitive abilities – here providers will share decision making with their caregivers) etc.”* [Quebec model]

Besides their different segmentation, coordination and engagement structures, the models analyzed use a broad range of outcome measures. Mainly the Canadian programs (Community agency lead model, ICCP and Community Health Centre in Ontario, and all cases in Quebec) collect a relatively extensive amount of data on health outcomes, patient and caregiver experiences and costs. For example: the community agency lead model, collects data on service utilization, client experience/satisfaction, ER visits and fall rates, quality of life as well as a variety of primary care measures. Other practices measure their success in a less standardized way, for instance by focusing on process measures (NZ Network model) or by using more pragmatic and informal measures (South Holland). Three programs reported that no outcomes are identified or systematically measured. Only one program (representing the three Quebec cases) reports that several formal research studies have been conducted for the evaluation of the model.

### Comparing the policy environment: governance, funding, staffing, innovation

The 11 cases analyzed had various governance structures. Most models had a shared governance structure consisting of partnerships between organizations involved in the continuum of care for their target populations (South Holland, Utrecht Hills, the Community agency lead model, NZ Network model). Partner organizations were often represented in steering committees of directors which included partner representation. Other programs were led by a single organization operating with a board of directors (ICCP, Community Health Centre). The Quebec program (representing 3 cases in different sized jurisdictions) follows a fully integrated model with the structural merger of all health and social care organizations under a single governance structure.

Funding approaches also varied across models. The Maori health organization model was funded by multiple sources – government, district health boards and primary health organizations. South Holland and Utrecht Hills adopted mixed funding models through local/municipal governments and private health insurers. Other models had dedicated funding through partnerships of organizations for specific staff within a primary health care clinic. For instance, the ICCP model was jointly funded (in-kind) with staff supported by the primary care practice and the local community agency. The Quebec model is based on a global budget to a single governance structure financed publicly through taxation.

Although multidisciplinary team-based care was an essential component of each case, most staff stayed employed by their mother organizations. Two approaches emerged on the staffing models that ensured multidisciplinary team-based care. First, South Holland, Utrecht Hills and NZ Network programs did not change their staffing models – these programs focused on changing professional attitudes towards improved inter-professional collaborative relationships. Second, other programs opted for co-location of staff. For instance, the ICCP model co-located community care coordinators to multidisciplinary primary care teams while the Quebec model co-located nurses and social workers to community-based family medicine group.

While nearly all cases used some sort of IT system to store and share data, our analysis reveals models have two main data sharing issues in common. First, the models faced challenges in linking data between the “newer” IT systems and the “older” IT systems. In fact, the newer IT systems were often layered upon existing IT systems. Furthermore, older technologies like faxing were still used to share data across organizational boundaries. Second, there was a lack of interconnectivity between IT systems of various health and social care providers. For instance, in some programs, the IT systems of nurses, social workers or community-based family physicians were not inter-connected. Co-location of staff in the ICCP model facilitated data sharing because community care coordinators could access the IT system of their primary organization and share relevant data with their primary health team. A challenge related to the use of IT by different professionals is the access to data entry compared to reading only. This had an impact in the interdisciplinary communication.

Innovation was an important aspect of the programs we analyzed. We identified several local care delivery innovations across the programs. Most programs endeavored to assign a single contact person responsible for the coordinating health and social services for a user. New professional roles like the care navigator were developed in the Maori health organization model. Co-located hub sites that brought together different professionals from different organizations was an innovative feature of the Community Health Centre model. The Quebec models developed innovative and comprehensive multidisciplinary health and social care evaluation tools (such as the OEMC (*outil d.évaluation multiclientèle*) tool) that facilitated inter-professional collaborations.

## Discussion

The results presented here represents a step in the development of an international standard for reporting integrated care initiatives, offering a cognitive test and additional validation of the Case Template developed to describe integrated care cases. We have demonstrated that the Case Template can successfully be applied to disparate international research studies, generating comparable data across 11 cases from 3 different research programs across 4 countries. In this discussion, we suggest modifications to the Case Template based on this work, and identify potential value this approach brings to different stakeholders, with an emphasis on value for adopters of integrated models.

### Challenges adopting the Case Template

Based on our application of the template as well as feedback from the ICIC19 workshop, we identified the following adoption challenges:

*Definitional clarity:* In particular during the workshop, delegates struggled with definitional clarity needed to help them apply their experiences and models to the Case Template. One notable example provided by a delegate was around the concept of a “care or patient navigator.” This term was not consistently used across different jurisdictions amongst delegates, nor was it used consistently in the iCOACH and Vilans cases, leading to an in-depth discussion of what is meant by navigation as compared to coordination. It was determined that key terms in the template would need to be well-defined to ensure clear understanding and comparability across jurisdictions.

*Attending to perspective:* Another important reflection in the workshop discussion was regarding attending to who exactly would be filling out the templates should these be implemented across multiple jurisdictions and programs looking to describe their models of care. It was noted that a front-line clinician and executive-level manager of the same model may respond to the same questions differently, requiring that we be clear on who in the organization should be filling out the templates to ensure comparability across sites. Divergence in perspective from different stakeholders has been found to impede implementation of integrated care [34], and as such a critical component when thinking of scaling and spreading models.

*Redundant concepts:* Another area of struggle for the research team, as well as for delegates in the workshop, was in teasing apart concepts that felt too similar or event redundant. The most prominent example of this was in questions around eligibility in the segmentation section, and the intake process in the coordination section. It was found that often models of care would determine eligibility as part of their intake process via assessments, surveys, or interviews with patients and their families.

*Capturing culture:* Both the research team and workshop delegates noted that the Case Template captures more process-oriented aspects of integrated care with less emphasis on cultural practices that are equally important to driving models of integrated care[35]; one notable exception is a prompt questions regarding having a patient and family engagement culture at the organization. In research team discussions, as well as those in the workshop, it was found that we could not speak about what worked functionally without attending to normative issues of relationship and culture that were considered necessary to make processes work. Even in filling out the templates, jurisdiction leads would often include reflections on these normative aspects of integrated care as they could not be removed from the processes being described.

*What level of context details matter:* A consistent debate amongst the research team, and reflected in workshop discussions was the level of detail required in filling out the Case Template. This was particularly important with regard to sharing learning on how cases addressed common issues. For example, when discussing the differences between funding models, it was important to drill down on key details such as navigating union agreements and how to engage multiple funders so cases could learn how to navigate these difficulties. Other challenges, however, required less detail to understand across cases. In discussing inter-professional teams, it was determined to be less important to know exactly how an inter-disciplinary team was structured (eg, how many physicians, nurses, or social workers involved) or communicated, than it was to understand how the team built their relationship so they could work together to meet patient needs.

While challenges were noted, the delegates at the workshop generally felt the structure of the template captured key aspects of integrated care. It was clear in the discussion that the template was not considered to be a stand-in for more rigorous comparative case study research methods, but rather is most useful as a practical tool to describe cases and support knowledge sharing across boundaries. The participants felt the relevance of the framework was to summarize case studies and initiate a conversation to share learning on key features of integrated care models.

A critical learning was that we were successful in adopting the Case Template given the team’s research skills and in-depth knowledge of cases. While this allowed us to create comparable data sets, this may not be easily applied by managers who wish to describe their models of care. As such we offer two modifications to the template. The first is a refinement of the template that can be adopted by other researchers seeking to use the template to compare disparate empirical cases of integrated care. The second is a simplified template that we anticipate can be more readily adopted by managers to quickly describe their model in a standardized way.

### Modifying the template for researchers and the value of the approach

During the post-workshop discussion, the research team identified the key areas where the template required modification based on: 1) what was discussed at the workshop and 2) notes and minutes from analysis meetings in which the challenges of applying the framework across cases were documented. We determined that many of the challenges identified in our application of the template and workshop with delegates from ICIC19 can be mitigated by modifying the template as well as providing clear definitions and guidelines for its application. Much of the content and structure worked well, and will be strengthened through the following changes:

1. Reframing segmentation questions to focus on general population of interest for the model of care, maintaining the first question as is, and moving the referral question to be a part of the intake section under coordination.
2. Streamlining the prompts to reduce redundancy in questions.
3. Adding prompts to the segmentation, coordination sections, and part 2 of the template to capture normative aspects of integration (eg, relationships and shared values that underpin these processes).

To address the important aspect of perspective and definition, we also recommend adding:

1. Clear definitions of each concept (eg, care coordination) upfront, and a section where respondents can define concepts specific to individual cases as needed.
2. A section where individuals filling out templates can identify their role in the organization.

Finally, we recommend restructuring the approach to improve feasibility of use for secondary data analysis, allowing data to be extracted from available sources rather than using an interview format. We added an introductory page which addresses how to do this work, the issue of describing contributors, and a space to provide a high level context summary of factors viewed as influential on the model described (addressing the identified issue of context). We reflect these changes to the template in Supplementary Materials #2.

For researchers, this template can be used to determine comparability of case study data as a preliminary step before engaging in more rigorous comparative case study work. One approach to comparative case studies suggestion by the WHO is to look at available data with an aim to adapting it to a common unit of comparison [7]. Our proposed modifications to the Case Template can help to achieve this aim, and serves to address three identified challenges when engaging in service level comparisons across regional boundaries [16]:

1. Securing comparability in terms of key concepts as different regions may assign different meanings even to terms that are widely used. In particular be cautious when creating typologies which can often trade-off accuracy for simplicity. Including definitions and areas where definitions of key concepts can help address this challenge.
2. Attending to both *between* and *within* system-wide differences that may influence which contextual factors are at play. Regional-based differences need to be attended to, and so descriptions should be careful not to generalize one program description to an entire nation, particularly when looking at decentralized models of care delivery. The second on policy context offers a means to tease these differences apart.
3. Finding and selecting data that is able to be compared across disparate cases. A balance must be struck between comparing aggregate level data, without losing important context and nuance unique to individual cases. This is particularly challenging when comparing in-depth case studies which are rich, detailed and contextual. The proposed template points to key constructs and leaves room for different levels of detail descriptions as determined necessary by those applying the method.

The proposed modified template can help research teams describe cases including both program and contextual policy-related factors. For non-researchers, further simplification and standardization is useful.

### Simplifying the template for managers and providers

Keeping the modifications above in mind, as well as what was learned in applying this method, it is clear that our success in using the Case Template to compare and contrast a highly varied set of programs may likely be derived from having: 1) strong research backgrounds; 2) expertise in the area of integrated care; and 3) in-depth knowledge of the cases we were describing. There have been attempts by the researchers who developed the Case Template to have front-line managers and providers use it with much less success, mainly due to its depth and complexity. As part of this work to create a survey that could be used from front-line staff, research team members have been working with IFIC to review other survey tools alongside the Case Template to see if the tool could be simplified. These other tools were reviewed, and, alongside what was learned to modify the Case Template, a simplified template was developed. An initial version was written, then circulated to the team for review and discussion until consensus was reached. This second modification to the template is intended to be used by managers and providers working on the front-line to describe their cases. This simplified template can be found in Supplementary Materials #3. The intention here is to allow for a standardized approach to describing models of integrated care internationally that can be collected quickly and effectively directly from those delivering the model; reducing the need for the resource-intensive approach that relies on research teams.

A simplified, standardized template has value to many stakeholders in the system but, in particular, organizations seeking to provide innovative integrated care either by modifying their existing care delivery or adopting innovations that others have developed. In both circumstances, there is a need to accurately document or describe the innovation and to systematically understand which components or processes have been kept the same and which have been modified. These descriptions help organizations to more clearly see the main components of integrated care models, compare their existing ways of working, and see the path towards a more mature system (eg, moving from having no structured protocols for coordination processes, towards having clear protocols and strong commitment).

In the background section of this paper we presented Horton and the Health Foundations argument for the need to balance the “tightening” and “loosening” of program sections to support adoption of complex interventions [4,25]. Particularly in the context of adoption of complex integrated care innovations, there is a tension between having a very detailed definition or codification of the innovation that allows for fidelity and assurance of expected outcomes and allowing for modifications to take into account local context and resources [36]. The goal then is to find a “middle” way between descriptions that are too tight to be successfully replicated in new settings and too loose to allow for a reasonable expectation of predicted impact. Some recent work has shown that frameworks that are acceptable for descriptions of randomized trials may not be detailed enough to allow for meaningful spread and adoption [37]. We hope to test our new framework in the context of supporting adopters to determine if it is closer to the middle way than other existing tools [38].

A final value-add of the both modified and simplified templates is the opportunity to build a community of practice around the implementation of integrated care internationally that not only consists of those studying integrated care, but those engaging in it as well. Establishing continuous learning and social networks create opportunities for training and knowledge exchange that are found to be critical factors in supporting scale and spread of health system reform efforts [39]. We intend to use the simplified template to support sharing of knowledge, enable self-assessment, and help build social networks to advance scale and spread. First, we will pilot the simplified template at ICIC20 in Croatia with attending delegates, as well as through IFIC and its affiliate branches in Canada, Ireland and Australia with the longer term vision of generating a summary data set of integrated care models worldwide. The summary data set represents important shared knowledge that can be used by providers and managers to compare themselves to other models working in similar contexts. As IFIC already has a wide international member-base, it can also help facilitate additional social networking between models with similar profiles which can help support teams to come together across borders and then ask more detailed and granular questions to deepen learning and support scale and spread.

### Limitations and Future Work

To conduct this comparative model of integrated care, the team worked with data already collected through case study research. As there was no ability to probe beyond the information already available, some details regarding descriptions of the models may have been missed. We additionally were unable to determine, at this stage, the “correct” or “optimal” level of detail required to provide more granular guidance. The discussion at the conference offers some indications that focusing on higher level context variables offers insightful information to compare cases, and may be more feasible than providing in-depth detail at all levels. However, we recognize that this approach may miss some micro level differences that could be important for adopters and researchers to consider. More work to tease apart the “right” level of context detail is likely still required.

We also recognize the issue regarding differing perspectives of management and front-line staff that was raised at the workshop may be a substantive one, potentially signaling issues with culture and leadership approach of a model. As these are complex challenges we do not recommend unpacking them using a descriptive template such as is presented here. Instead identification of disparate perspectives within a single model may signal the need for researchers to dig more deeply, and for models to attend to misalignment in the understanding of the programs vision, aims, and processes amongst staff.

The sample of cases we chose for this analysis was necessarily based on a convenience sample of the studies we had already conducted. An application of our method to other cases may yield additional insights on the template, and as such we recommend the modified and simplified templates be viewed as “living documents” to be revisited and refined as they get applied and new insights are generated.

Finally, the two modified versions of the survey require further validation and testing, in particular, the simplified version needs to be tested with front-line providers and managers to ensure that it can indeed be easily applied and provide implementation guidance. As previously noted we intend to pilot the simplified survey in 2020 through IFIC, as well as at ICIC20 as a step towards further validation.

## Conclusion

This paper demonstrates that a standard case description template can be effectively applied as a secondary data extraction method; helping to create comparable descriptions of integrated care cases across international boundaries by drawing on data collected as part of case study research. The presented modified and simplified templates address a number of the challenges identified by the researchers in applying the tool and providers and managers who were presented the tool via a workshop at ICIC19. As demonstrated by the work presented in this paper, the modified tool will be valuable to researchers studying integrated care across different jurisdictions as a means to provide a high level comparable summary of key components of integrated care models. The presented simplified tool, we feel, has significant potential to be valuable to adopters of integrated care by offering a simple tool that can be used to summarize and compare cases, helping models to situate themselves as compared to peers, and make meaningful connections to other models as a means to further efforts to scale and spread models towards broader health system transformation.

## Additional Files

The additional files for this article can be found as follows:

10.5334/ijic.5413.s1 Appendix A. Full case descriptions.

10.5334/ijic.5413.s2 Supplementary Material 1. Integrated Care Case Study Descriptive Template Structured Interview Guide – used for Commonwealth Fund study.

10.5334/ijic.5413.s3 Supplementary Material 2. Integrated Care Case Study Descriptive Template – Modified Version.

10.5334/ijic.5413.s4 Supplementary Material 3. Integrated Care Case Study Descriptive Template – Simplified Version.
