# Supplementary material for: Comparing International Models of Integrated Care: How Can We Learn Across Borders?
Source: Int J Integr Care. 2020 Apr 1;20(1):14. doi: 10.5334/ijic.5413 (PMC7147684; doi:10.5334/ijic.5413)
Supplement: Appendix A. — Full case descriptions. [file ijic-20-1-5413-s1.pdf]

Appendix A: Full case descriptions

Case	Segmentation	Coordination	Engagement	Success measures	Policy Context
**SUSTAIN**					
South Holland	***Target group:*** frail, multiple health and social care needs (but broadly defined)<br><br>***Entry points:*** self-referral (clients and families) or by professionals in the community (prevention driven with active community communication)	***Intake***: Conducted by any provider using a standard tool (Self-reliance matrix) filled out during a home visit (using a tablet). Patient then assigned navigator (anyone on the team, all trained for this role – likely with most experience with care needs of patient).<br><br>***Primary care providers***: Each team includes community nurse or GP practice nurse. Direct connection to GP practice varies.	***Patient engagement***: A strong belief of the teams, but not formalized. Also engagement is limited due to the low functional status of clients.<br><br>***Patient self-management:*** Similar to issue above, believed to be important but difficult to operationalize. Additional challenge of different characteristics of neighbourhoods.<br><br>***Caregiver engagement***: Not yet a clear component	***Maturity:*** Program admitted first client in 2015 and has served 5,000 since. Served approx. 300 in the past 6 months. Program started as a pilot (3 teams) and scaled up in 2016 (27 teams). Composition, objectives and aims of teams varies by neighbourhood.<br><br>***Measures:*** Better health outcomes, patient/caregiver experience and lower costs – these are not formalized in measures (not unusual).<br><br>***Data collection***: No data on program activities are collected. Currently developing performance indicators.<br><br>***Evaluation:*** No formal evaluation conducted.	***Financing for model:*** Both municipal (public tender and subsidized funds) and health insurer financed.<br><br>***Staffing model:*** All professionals stay employed by their mother organizations. Next to their daily work, they get extra hours for doing the multidisciplinary work/meetings. Professional training is executed by the local (applied) university and funded by the municipality.<br><br>***Governance structure:*** Shared governance model. All involved parties (health and social care providers, GPs, municipality, health insurers) are represented in a steering group. However, the two financing institutes (insurers and local government) are directing. No performance data is collected yet.

		Integration: At minimum social worker, community nurse and municipal social care worker. Can add: dementia case managers, physicians, social housing, etc…<br><br>***Transitions:*** All providers still linked to their parent organizations which can facilitate transition<br><br>***Information sharing:*** All providers can access a shared data platform which includes online communication tool (all teams trained on it).	of strategy – but family issues captured as part of the assessment process. Experimenting with digital tools to support caregiver engagement (interest in building this long term).		***Health and social care data sharing structure:*** To facilitate data linkage, a shared IT system has been developed. However, 'old' systems are still being used. Administrative burden is a risk.<br><br>***Care delivery innovation:*** Most innovative part is that a person/family has 1 contact person, and that integration takes place in all phases of the process: from intake to care delivery.
Utrecht	***Target***	***Intake***:	***Patient***	***Maturity:***	***Financing for***

Hills	***group:*** Clients with dementia living in the community (requires GP diagnosis) to have day care and caregiver support.<br><br>***Entry points:*** Case manager or POH elderly care (assistant to the GP focused on elderly care) can refer to the program via application. Client can also reach out to centre upon recommendation by case manager and/or POH.	Once referred a social team member meets (home visit) with the client and deems eligibility (to receive reimbursement from the Law on Social Support). There are guidelines but no clear assessment criteria or rules for eligibility. Daycare centres can also decide if they allow the client to enter.<br><br>***Primary care providers***: First contact with GP to gain diagnosis. Case manager is the responsible to guide and support client in their home and can link	***engagement***: Not a clear component of strategy, but all providers try to involve preferences and needs of client and informal caregiver. No training in shared decision-making.<br><br>***Patient self-management:*** No clear strategy. Social team will look at what the clients can do.<br><br>***Caregiver engagement:*** Network includes an actor focused on informal caregivers that provides information, advice, guidance and support. Support for caregivers actively included.	Program started in 2011 and still at a pilot stage. Network directors still actively looking for funding to keep the program going.<br><br>***Measures:*** Outcomes not monitored.<br><br>***Data collection***: Outcomes of the activities not currently monitored – although still viewed as important by network members.<br><br>***Evaluation:*** No formal evaluation conducted. (SUSTAIN interviews considered first form of 'evaluation')	***model:*** Funding comes from two sources: local government (both public tenders and subsidized) and health insurers.<br><br>***Staffing model:*** No changes in the staffing model. The program contains a new attitude towards working together with other professionals/organizations.<br><br>***Governance structure:*** Shared governance model with a steering group of directors of the three largest care and cure organizations, an alderman of the municipality, a GP representation and Alzheimer Nederland (client representation).<br><br>***Health and social care data sharing structure:*** No data sharing structure.<br><br>***Care delivery innovation:*** One easily reachable contact person (case manager) for the client .

		to additional services as need increases. Primary care participates in multi-disciplinary team meetings.<br><br>***Integration:*** Dementia services network in Utrecht Hills include a wide array of professionals and organizations specializing in all phases of the disease. Includes a steering committee and operational workgroup (those delivering care, 23 individuals) who will engage in multi-disciplinary team meetings and will			

		refer to each other as needed.<br><br>***Transitions:*** Transition protocols at organizational level only.<br><br>***Information sharing:*** Not allowed to share information electronically across organizations and professionals (no platform available). Will rely on multi-disciplinary meetings occurring every 6 weeks			
**ONTARIO**					
Community agency lead	***Target group:*** Anyone in need of services but primarily older adults requiring health and social care services.<br><br>***Entry points:***	***Intake***: Informal process which varies from program to program. Can self-refer or be referred within and outside of the organization	***Patient engagement***: While client/person-centred care is a core aim, there is no formal support or training. Care coordinators and allied	***Maturity:*** The Community agency is an established organization with ongoing funding at its initial site. It's growing but not seeking to replicate itself.<br><br>***Measures:*** The Community agency collects	***Financing for model:*** Working within existing funding structures in Ontario, the Community agency is able to bring health and social care financing under a single organizational roof allowing for some operational cost sharing.

	Client can self-refer to the organization. Some programs have established eligibility criteria (usually for government funded programs).	.<br><br>***Primary care providers***: Involvement dependent on program, many can function without primary care connections but may require reaching out for follow-up. Programs run in the Family Health Team are often done with nursing and social work staff, physicians don't need to be involved but can easily refer patients and communicate with provider running programs to follow-up.<br><br>***Integration:*** Many examples of interprofessional teams	health do report engaging in collaborative processes with patients, including goals-of care discussions. Patients cultural background sometimes precludes this process (they do not expect to be part of decision-making).<br><br>***Patient self-management:*** Several programs strongly support patient self-management, in particular an emphasis on empowerment and responsibility. Chronic Disease Management Programs run out of the primary care team does this as	data on: service utilization, client experience/satisfaction, ER visits and fall rates, quality of life, primary care measures (for primary care as dictated by the regional body, like wait times, follow-up post discharge, and patient vitals and symptoms specific to certain programs (e.g. grip strength in the geriatric program)<br><br>***Data collection***: While there are a number of measures listed they are not routinely collected (expect for the primary care team which needs to collect measures for the regional reporting). Data collection done mostly as needed to inform strategic planning and resourcing decisions.<br><br>***Evaluation:*** There has been	***Staffing model:*** The innovation is in bringing multiple types of health and social care service providers under a single organizational umbrella. In their respective program areas (eg, transportation, primary care) the models is similar to similar organizations in Ontario.<br><br>***Governance structure:*** As a non-profit, Caregiver has 3 boards of directors, one for each social/community care, primary care (the family health team), and the foundation of the community agency. Unique here is the opportunity for the health and social care boards to work together in their planning. Boards review performance data most often aligned to reporting requirements from multiple funders (Ministry, regions, charitable organizations)<br><br>***Health and social***

		but not necessarily formally coordinated. Some programs include a more formal coordination model, but outside these programs coordination is managed by multiple care coordinators working in different programs and units – unclear who is most responsible should a client have multiple coordinators.<br><br>***Transitions:*** There is only one formal program, Assess and Restore, that is used to help transition individuals from hospital to the community	well. Educational materials also emphasized for patients.<br><br>***Caregiver engagement:*** Caregiver engagement is not formalized. Mainly caregiver support is through the respite program.	no formal evaluation done.	***care data sharing structure:*** Similar to the rest of Ontario. The health and social care branches each have their own information systems that do not integrate. Still using fax and calls to share data across those boundaries. Even greater challenges when sharing information with external partners.<br><br>***Care delivery innovation:*** Vision of integration through bringing multiple services together under a single organizational roof serving a particular population (Primarily Chinese older adults and other immigrant populations in the area) .

		(6 week program)<br><br>***Information sharing:*** The Community agency has two data platforms, one for the Community arm and the primary care EMR systems. Patient data also sits on the provincial homecare repository CRIS for patients getting home care services. Most communication pathways are generated as needed.			
Integrated Client Care Program (ICCP) – partnership model between primary care and home care	***Target group:*** The Integrated Client Care Program (ICCP) focuses on the top 1-5% frail older adults in need of integrated	***Intake***: Intake depends on the RAI evaluation (see target group). The Care Coordinator from the CCAC typically	***Patient engagement***: Patients engagement occurs at this site and is an increasing focus. There are patient and family carer seats	***Maturity:*** The ICCP program began in 2012 and is a replication from the ICCP program in palliative care run out of the CCAC. Other FHT programs like Virtual Ward	***Financing for model:*** The ICCP program is funded by the FHT and CCAC through paying for specific staff to run the program. For the FHT the staff is now part of the global budget.

	services. Patients are assessed up the RAI tool.<br><br>***Entry points:*** Patients can enter the program through multiple entry points including the Family Health Team (FHT), the Community Care Access Centre (CCAC - government agency that connects patients to home care services), and through other partners and community agencies aware of the program.	takes responsibility for ICCP patients<br><br>***Primary care providers***: All patients on the ICCP program have sustained access to their primary care provider who is supported through a multi-disciplinary team<br><br>***Integration:*** There is a high level of professional integration with the multi-disciplinary team, as well as organizational integration between the FHT, the CCAC and hospital.<br><br>***Transitions:*** There is a formal program	on committees and strategic planning groups.<br><br>***Patient self-management:*** Collaboration and patient goal-setting is a part of the culture at the FHT and embedded into the ICCP program.<br><br>***Caregiver engagement:*** Caregiver support less formalized, but providers are attentive to caregiver needs and attempt to provide supports when they can. Not a formal process.	and IMPACT are also established and support the integrated model. IMPACT is a replication from another site.<br><br>***Measures:*** Standard FHT measures apply to the FHT for reporting to the LHIN on performance. It is noted by decision-makers that other measures are currently missing, but they would anticipate that reduced hospitalizations and ER visits be among their key measures.<br><br>***Data collection***: Data not available<br><br>***Evaluation:*** ICCP was not formally evaluated at the time of data collection. A different Virtual Ward program at Women's College has had a formal evaluation, as has the IMPACT program in other	***Staffing model:*** Unique staffing model which collocates the community partner (home care coordinator) in the multi-disciplinary primary care team to improve coordination and information sharing.<br><br>***Governance structure:*** The FHT, like other FHTs in Ontario has a board of directors that reviews performance metrics aligned with Ministry reporting requirements.<br><br>***Health and social care data sharing structure:*** Some innovative data sharing between the FHT and the local hospital (sharing medical records), electronic referral and information sharing with Toronto EMS (paramedics), and colocation of staff enables seeing health and social care data while in the primary care clinic.<br><br>***Care delivery innovation:*** The

		with the local hospital, Virtual Ward, which supports transitions for clients going from hospital to home. This is a clear process and protocol but only for patients at the local hospital – if patients end up in another hospital there is no process.<br><br>***Information sharing:*** Partnering organizations have connecting information systems (hospital and FHT), or individuals able to access multiple platforms (embedded care coordinator can see FHT		settings.	colocation model of ICCP, along with the virtual care and home visiting programs are innovative practices in Ontario.

		and CRIS systems)			
Community Health Centre	***Target group:*** The Community Health Centre model focuses on caring for individuals in the local community. Any individual with or without identification can access services when needed.<br><br>***Entry points:*** Clients can self-refer to any programs at Unison, subject to availability and wait-lists. Referral to programs is generally open and individuals can enter multiple ways.	***Intake***: Intake is most often managed by social workers and case managers, particularly with regard to more complex patients populations<br><br>***Primary care providers***: Clients will have regular contact with primary care providers and case manager and other allied health staff can easily connect to primary care providers as they are collocated. Often conversations are informal "hallway" conversations about clients.<br><br>***Integration:***	***Patient engagement***: The emphasis of the Community Health Centre model is on client engagement. Shared decision-making, collaboration and empowerment are central to the model. Unclear whether this is formally trained, but many providers (like social workers) will already have this orientation.<br><br>***Patient self-management:*** Emphasis is on empowering clients towards self-efficacy. Again, this is part of	***Maturity:*** Formed by a voluntary merger of two Community Health Centres (merged in 2006).<br><br>***Measures:*** Standard Community Health Centre measure will be collected and reported to the LHIN, and to their accreditation body. Measures in the Quality Improvement Plan include: screening and preventative measures (e.g. Pap tests), satisfaction/complaints, person-centred decision-making, 7 days post discharge follow-up, same day/next day appointments, resource measures (MD/NP backlogs),<br><br>***Data collection***: Data collection occurs quarterly on the above	***Financing for model:*** Funded as all Community Health Centres are funded in Ontario which includes a mix of global budget from the region and program funds available here and there through other sources.<br><br>***Staffing model:*** Multi-disciplinary primary care model with co-located social services. In some cases co-location is with partner organizations.<br><br>***Governance structure:*** Like other Community Health Centres, this one has a board of directors. Reviews reporting aligned with requirements from funders (mainly the LHIN/Ministry)<br><br>***Health and social care data sharing structure:*** Similar to other Community Health Centres they have a single EMR system that all Community Health Centre providers can

		The Community Health Centre includes a multi-disciplinary team as well as many health and social programs to meet the diverse needs of their community. They also partner with multiple local community organizations to meets needs (e.g. legal services, new immigrant services, local schools)<br><br>***Transitions:*** There does not seem to be a formal protocol for transitioning clients, but some referral pathways to partner organizations exist.	the Community Health Centre culture.<br><br>***Caregiver engagement:*** No formal caregiver process in place.	measures as part of their quality improvement plan.<br><br>***Evaluation:*** No indication of a formal evaluation beyond quality improvement work.	view. No integration with other systems. Data collected is primarily medical record data.<br><br>***Care delivery innovation:*** Innovative co-located hub model where they share space with community partners to improve accessibility for clients to other local services.

		Heavy reliance on informal communication between programs.<br><br>***Information sharing:*** Unison has a single EMR system that can be accessed by any Unison provider.			
**QUEBEC**					
	Target group: Functional Autonomy Measuring System (SMAF) used to determine eligibility – need a particular score to be included.<br><br>***Entry points:*** Patients with 2 or more YES answers on PRIMSA-7. SMAF is managed by a specialized team at a single point	***Intake***: SMAF scores guides a multidisciplinary care plan. Host organization and local organizations may have some flexibility in what is provided. Those with a SMAF >5 receive a case manager through home care services unit.<br><br>***Primary care providers***:	***Patient engagement***: Personalized care plan but shared-decision making difficult to operationalize. Culture of shared decision-making supported by government and leaders.<br><br>***Patient self-management:*** No clear self-management support aspects of program	***Maturity:*** CLSC's operational since 1970s with 100,000's since than. It is an established government run program with secure funding and spread across the province.<br><br>***Measures:*** Better health outcomes, patient and caregiver experience and lower costs. Related to government healthy aging policy with specific indicators: reduced wait times, reduced ED visits, #	***Financing for model:*** Public fund troughs taxation. In complementary, patient may directly pay for services from community agencies that are mostly not covered by the public insurance.<br><br>***Staffing model:*** All professionals stay employed by their mother organization. Recent initiatives are in place to "lend" allied professionals (nurses, social workers, dieticians etc.) to privately owned Grouped Medical practices – the allied professional are still employed by the

	of entry for defined geography. Clients can also self-refer	Some regular contact but challenging to connect to primary care as they are privately owned. Case managers have primary responsibility.<br><br>***Integration:*** Types of services offered varies by local organization but all include primary care in the community, acute and surgical, home care, nursing home, supportive housing, community day care and social supports. Some co-location but not in all sites.<br><br>***Transitions:*** Some	***Caregiver engagement:*** Some caregiver supports offered (e.g. respite days) – no information regarding formal training.	clients in the program.<br><br>***Data collection***: Performance indicators reported on regularly.<br><br>***Evaluation:*** Several formal research studies conducted to evaluate the model. Developed OSIRSIPA tool to monitor implementation and outcomes.	mother organization but work in private physician clinics. Family physicians in the community are paid through public insurance but are autonomous workers.<br><br>***Governance structure:*** *Since 2015,* Almost a full integration of public establishment under the same governance (hospital, rehabilitation, home care, long care term facilities) Vertical governance structure. The HSSCs are public health and social care agencies that are mandated by the government to organize care delivery in their territories. The HSSCs have to lead in establishing local joint governance boards for various health problems with their local partners in the community (physician clinics, nursing homes, private community agencies etc.).<br><br>***Health and social care data sharing***

		organizations have dedicated care transitions provider (engage in pre-discharge meetings)<br><br>***Information sharing:*** Have ICT systems to facilitate integration and transitions, in particularly tools that send transfer information electronically. Government mandated (RSIPA system). Some variation in access due to location.			***structure:*** There is a government mandated IT system (the RSIPA) that is shared between various agencies within the HSSC. However, some private agencies do not have access to this public IT system. Furthermore "older" IT systems co-exist with the public IT system.<br><br>***Care delivery innovation:*** Introduction of several initiatives. E.g. formalization of care coordination by case managers, use of multidisciplinary individualized service plans, and use of multidisciplinary health and social care evaluation tools.
**NEW ZEALAND**					
Primary Health Organization – home visiting program	***Target group:*** Targets support to 5%of the local populations (12,000) deemed most in	***Intake***: Clear referral pathway from the PHO clinics, although eligibility is unclear.	***Patient engagement***: There is strong patient and family engagement focus of the model. The impetus for	***Maturity:*** At the time of data collection the program was spread from pilot to a sustained model, in place for 2 years.<br><br>***Measures:*** No	***Financing for model:*** PHOs pay for the community nurse and worker.<br><br>***Staffing model:*** New staffing model in which two providers go out into the community to

	need of "extra support". Emphasis is on serving Maori population, although all clients can be referred.<br><br>***Entry points:*** Teams get referrals from one of 6 Primary Healthcare Organizations in the region.	***Primary care providers***: There is limited involvement by the primary care providers in the specific program. Mainly the service is run by a community nurse and community worker (kaiwaihna). There is limited involvement beyond referral.<br><br>***Integration:*** There are a limited number of providers, mostly the program is run by the nurse and community worker. They can, however, access services at the PHOs as needed. And social support is built into	referral is to improve patient engagement specifically.<br><br>***Patient self-management:*** Linked to the engagement piece, self-management and building self-efficacy are linked to the engagement process.<br><br>***Caregiver engagement:*** Similar to patient engagement, family engagement is a focus for this model of care. Support is provided for them as for clients.	specific program outcomes identified. General reliance on case stories<br><br>***Data collection***: Not routinely collected<br><br>***Evaluation:*** No formal evaluation	deliver services and improve patient engagement.<br><br>***Governance structure:*** PHOs reporting to the DHB – same as the model before but they report on this program now as well.<br><br>***Health and social care data sharing structure:*** The six teams of community workers are able to input and see patient data from the 6 PHOs they serve<br><br>***Care delivery innovation:*** Changes in who directly cares for patients (community providers), stronger involvement of patients and families in decision making (Whanau Ora tradition), and improved data sharing via community workers.

		the model of care (provided by community worker directly or by linking to other services).<br><br>***Transitions:*** No structured transition model existing. Primarily a community oriented program.<br><br>***Information sharing:*** Client data resides in the PHO EMR systems. The community pair is provided access to the EMRs at the clinic or remotely. They can also access system utilization data through another portal (e.g. see hospitalizati			

		ons and ED visits)			
Maori health organization	***Target group:*** The Maori health organization trust serves a geographically located population without clearly established eligibility requirements for receiving services.<br><br>***Entry points:*** Referrals can occur from multiple programs, and patients can self-refer. Process seems to vary from program to program – similar to Ontario Community Health Centre model. Shared EMR system allows for	***Intake***: Clients can make appointments with whatever services they need by calling the central office. Providers get back to clients to schedule appointments. Some outreach occurs for some high risk patients.<br><br>***Primary care providers***: There is limited involvement. Providers in the trust reach out to client GPs, but they don't work directly together.<br><br>***Integration:*** With the exception of GPs, there are a wide variety of	***Patient engagement***: Client engagement is a focus for this model of care with a number of programs designed specifically to support engagement (chronic disease management and mobile nurse unit). Goal-setting and care planning occur regularly with clients.<br><br>***Patient self-management:*** Similar to the Primary Health Organization home visiting case, Whanau Ora culture is being the model which is intrinsically drives a self-efficacy	***Maturity:*** The trust was established in 1999, with 6500 clients/year. This is an established program with ongoing funding, but does not seem to be replicated elsewhere.<br><br>***Measures:*** Unclear what the overall program goals are and what measures are included.<br><br>***Data collection***: Managers report having to report on metrics (but these are not specified). It's viewed as reporting against a contract.<br><br>***Evaluation:*** The Trust engages in PDSA cycles to improve performance. Other formal evaluations not conducted.	***Financing for model:*** Funding comes from multiple sources (government, DHBs and PHOs). Established contracts to deliver non PHO services.<br><br>***Staffing model:*** Not necessarily new – more of a new partnership modell<br><br>***Governance structure:*** The Trust has its own governance structure including the CEOs of partner organizations<br><br>***Health and social care data sharing structure:*** Data linkage available across PHOs (see coordination section)<br><br>***Care delivery innovation:*** Relatively new model of care that is managed by care navigators that connect patients and families to needed services. Emphasis on Whanau Ora to ensure strong patient and family involvement.

	electronic referrals between services within the Maori health organization.	health and social and mental health care providers. There are also a number of chronic disease management groups and public health programs. Case management occurs at team meetings and is done by an NP.<br><br>***Transitions:*** There is a robust referral process to connect clients to mental health and specialist services in community and hospitals. Not necessarily a traditional "transition" service though.<br><br>***Information sharing:***	and empowerment approach. Formal training not mentioned specifically, but it is part of the model of care.<br><br>***Caregiver engagement:*** Similar to patient engagement and empowerment described above.		

		Data linkage system available between the PHOs in the networks to connect to the Trust. There is also a HealthLink system to support referrals. Not consistent across all organizational connections.			
NZ Network Model	***Target group:*** The DHB serves a broad population but the CREST and care coordination programs focus on 65 and older population transitioning home from hospital.<br><br>***Entry points:*** Clients access services through Liaison Nurse who	***Intake***: Assessments used by Liaison nurses and care coordinators to assign services based on function and need (eg, interRAI)<br><br>***Primary care providers***: GPs play an active role in NZ Network Model, referring patients as needed to programs	***Patient engagement***: Goal-setting part of care delivery (particularly for CREST programs), not part of DHB training but embedded in professional training and approach.<br><br>***Patient self-management:*** Area of focus particularly for the CREST program	***Maturity:*** New model in DHB established in 2006/7 but gained traction in 2011 post earthquakes. An established program with ongoing funding.<br><br>***Measures:*** Emphasis on process measures (early discharge), also collect patient satisfaction and engage in peer review meetings<br><br>***Data collection***: No regular reporting mentioned in interviews – but	***Financing for model:*** DHB shifted to activity-based payment model for hospitals and bottom-up focused alliance contracting where maximum collective gain can only be realised if all parties support one another and agree to share any losses<br><br>***Staffing model:*** Unchanged – what has changed is how they work together<br><br>***Governance structure:*** Shift towards a Network model reliant on partnerships and governed by

	identifies eligible individuals in the hospital. Referrals for case management and care coordination programs for older adults can come through GPs, other providers or through self-referral.	and following up with other providers. They will engage in case conference calls with other providers as well.<br><br>***Integration:*** Involves a wide range of health as social care services some of which are tailored to older adults with complex care needs. Providers regularly speak across boundaries to deliver care.<br><br>***Transitions:*** CREST is a structured transition program from hospital to home. Care coordinators and case managers work to help	with an emphasis on enablement and support.<br><br>***Caregiver engagement:*** Not an emphasis	likely occurring particularly for funded partners<br><br>***Evaluation:*** No formal evaluation to our knowledge	Alliance Support team.<br><br>***Health and social care data sharing structure:*** Not necessarily new but part of the NZ approach to data where patients have unique identifiers across health and social care data platforms to facilitate finding information.<br><br>***Care delivery innovation:*** Most notable shift is in moving clients out of hospital and into the community setting faster through partnerships with social care providers and enablement program (CREST)

		integrate other services. Teams across services also work together.<br><br>***Information sharing:*** Use a few systems to share information include CCMS, SAP, Momentum, Health Connect South and One Health Now. Providers can access patient data that sits on these systems from different settings (eg, pharma, labs, clinical care, hospitals).			
