# Supplementary material for: Comparing International Models of Integrated Care: How Can We Learn Across Borders?
Source: Int J Integr Care. 2020 Apr 1;20(1):14. doi: 10.5334/ijic.5413 (PMC7147684; doi:10.5334/ijic.5413)
Supplement: Supplementary Material 1. — Integrated Care Case Study Descriptive Template Structured Interview Guide – used for Commonwealth Fund study. [file ijic-20-1-5413-s2.pdf]

SUPPLEMENTARY MATERIAL 1

Integrated Care Case Study Descriptive Template Structured Interview Guide – used for Commonwealth Fund study

Part 1: Program Design -Instructions

Start the data collection for each dimension with a specific "prompt" question. The prompt question asks the respondent to set the program in context of how much the program is built around or invested that design dimension. The prompt question is followed by a set of more detailed questions that delve more deeply in the program inputs, activities and outputs. All respondents should be asked the prompt questions. The use of the more detailed questions can vary depending on the depth of that design dimension.

Part 1: Program Design		
Segmentation	Programs for high needs populations are often designed to identify specific populations that can be referred to the program.	
	Questions	***Prompts***
	#1: Which **one** of the following statements do you think best describes the eligibility or referral rules for the program?<br>a. No firmly established rules exist, definition of eligible patients variable<br>b. Some rules in place to guide patient selection, but some flexibility remains in defining eligible patients<br>c. Clearly defined rules in place to define patient eligibility	Prompt 1<br><br>If there are no firmly established rules, then how is eligibility for the program determined?<br>Do providers or programs managers make the eligibility decision?<br>Do patients or caregivers decide?<br>Are there plans to create rules in the future?<br>If not, why are there no clear rules?<br><br>Prompt 2<br><br>How were the rules defined?<br>Was the development of the rules driven by costs or quality issues?<br>Were the rules developed within the host organization or were they the result of priorities set by funders or by external accountability or performance measurement initiatives?<br>Were the rules developed by a multi-sectoral team?<br>Do they include both health and social care needs or characteristics?<br>Did patients and caregivers have a role in defining who should be eligible?<br>Can you provide me with a copy of the

		eligibility rules?<br>How much flexibility is there in the application of the rules?<br>Who makes the final decision about eligibility?<br>Is there an appeal process if someone is deemed ineligible?<br>Is there process to change the rules of eligibility and who approves any eligibility rules changes?<br><br>Prompt 3<br><br>How were the rules defined?<br>Was the development of the rules driven by costs or quality issues?<br>Were the rules developed within the host organization or were they the result of priorities set by funders or by external accountability or performance measurement initiatives?<br>Were the rules developed by a multi-sectoral team?<br>Do they include both health and social care needs or characteristics?<br>Did patients and caregivers have a role in defining who should be eligible?<br>Can you provide me with a copy of the eligibility rules?<br>How much flexibility is there in the application of the rules?<br>Who makes the final decision about eligibility?<br>Is there an appeal process if someone is deemed ineligible?<br>Can patients and caregivers appeal if they are deemed ineligible?<br>Can providers or managers appeal if a client is deemed ineligible?<br>Is there an audit process to determine if all clients were eligible on admission?<br>Is there process to change the rules of eligibility and who approves any eligibility rules changes?
	Program can use different strategies to apply referral or patient selection rules.<br><br>***#2:*** Which **one** of the	Prompt 1<br><br>How do new clients get referred to and admitted to this program?<br>Is there some form of communication between a

	following statements do you think best describes how the eligibility or referral rules are used in this program?<br>1. There is an informal process for referring people into the program?<br>2. A care provider has a clear procedure for identifying or referring patients based on rules<br>3. The process for identifying or referring patients is built into a data system that alerts providers that patient is eligible	person who decides that they are eligible or might benefit and the person who takes them into the program?<br>Do clients enter the program directly with no referral process?<br>How do patients, caregivers or clients find out about the program?<br><br>Prompt 2<br><br>Do you keep track of how many patients referred to the program meet the eligibility rules?<br>Do you keep track of the number of individuals who are referred, are eligible, but not admitted to the program?<br>Is the variation in how providers make referrals to the program?<br>How do providers find out about the program?<br><br>Prompt 3<br><br>What is the data infrastructure that supports the alerts on patient eligibility?<br>If there is a data system for alerting, is there a process in place to see if there is subsequent enrolment?
Coordination	A common element of programs is single point or centralized process for intake or enrollment of patients in the program.	
	#3: Which **one** of the following statements do you think best describes the process for patient intake or enrollment?<br>1. No established individual or centralized process for intake of new patients<br>2. Clear process exists but implemented variably by different program navigators or health care administrators<br>3. Single or established	Prompt 1<br><br>Are data from the intake process collected in a standardized fashion?<br>Are data from the intake shared?<br>Is program intake and data collection part of the normal care process with no specialized data collection and no specialized or unique form of data sharing?<br><br>Prompt 2<br><br>Is there an individual(s) who is designated as responsible for majority of program intakes?

	group of designated patient navigator(s) responsible for intake of all new patients in structured manner	Can many different individuals provide program intake?<br>Do these individuals include both care providers and administrative staff?<br>Is there an individual who takes a comprehensive history and conducts a comprehensive assessment of health and social care needs?<br>Is there a single phone number that is used for access to the program by clients?<br>If there is a "patient navigator" role, then what is the training or profession of that individual?<br>When does that navigator start working with a patient – on enrolment, after a certain point in the care journey or on an as needed basis?<br>Is the intake process available quickly and/or on a 24/7 basis? What do you define as quickly? Within hours, days or weeks?<br>Is there a clear protocol for the assessments that are done at intake?<br>Is there a process to monitor the quality of the intake process?<br>How long does the intake process take?<br>How are data from the intake process collected?<br>How are data from the intake shared?<br><br>Prompt 3<br><br>Who is designated as responsible for majority of program intakes?<br>Can individuals provide program intake and also have other duties?<br>Is there an individual who takes a comprehensive history and conducts a comprehensive assessment of health and social care needs?<br>Is there a single phone number that is used for access to the program by clients?<br>How is the person in the "patient navigator" trained for that role?<br>When does that navigator start working with a patient – on enrolment, after a certain point in the care journey or on an as needed basis?

<table>
<tr><td></td><td></td><td>Is the intake process available quickly and/or on a 24/7 basis? What do you define as quickly? Within hours, days or weeks?
Is there a clear protocol for the assessments that are done at intake?
Is there a process to monitor the quality of the intake process?
How long does the intake process take?
How are data from the intake process collected?
How are data from the intake shared?</td></tr>
<tr><td></td><td>Many programs involve primary care providers (family physicians, general internists, geriatricians, pediatricians, nurse practitioners) as providers.

#4: Which one of the following statements do you think best describes the involvement of primary care providers in the program?

a. Limited involvement of primary care providers, including nurse practitioners.
b. Regular contact with at least one primary care provider, but primary care provider is not responsible for comprehensive management of patient needs through this program
c. Primary care provider clearly responsible for managing the care needs and care processes of the program’s designated patients</td><td>Prompt 1

If primary care has a limited role, then what provider group is the “most responsible” for care?
If primary care providers have a limited role in the program now, has that changed over time?
Is there a plan to expand primary care roles in the program in the future?

Prompt 2

What provider group is responsible for comprehensive management of patient needs?
How is regular primary care contact maintained?
Do primary care providers have any roles in activities such as
drug reconciliation and medication management
planning transitions in care
ongoing review of care plans
monitoring overall patient outcomes
structured multi-disciplinary care team meetings

Prompt 3

Is a primary care provider designated as the “most responsible” provider for the majority of the patients in the program?
Are specific designated primary care providers assigned to have patients of the program make up the majority of their care load or can primary</td></tr>
</table>

		care providers manage patients in this program as one part of a wider case load?<br>Do primary care providers take leadership roles in activities such<br>drug reconciliation and medication management<br>planning transitions in care<br>ongoing review of care plans<br>monitoring overall patient outcomes<br>structured multi-disciplinary care team meetings
	Programs often have a focus on integrating health and social care services.<br>***#5 Prompt Question:*** Which **one** of the following statements do you think best describes the integration of health and social care services in the program?<br><br>a. Programs involves a limited number of different health and social care provider types and is focused on specific social or health services or settings<br>b. Program involves multiple different health and social care provider types, care settings and organizations and provides both health and social care but not in highly coordinated manner<br>c. Program involves a wide range of health and social care provider types working in a coordinated system of delivery of integrated and comprehensive health and social care services in a	Prompt 1<br><br>What types of providers are involved in the program?<br>Nurses<br>Social Workers<br>Physician Assistants<br>Pharmacists<br>Behavioural supports through social work, psychiatry, or psychology<br>Secondary specialist care<br>Any other health human resources not yet described<br>What types of care settings and organizations are directly involved in the program?<br>Primary care in community<br>Inpatient acute medical care and surgical care<br>Home care<br>Nursing home<br>Supportive housing<br>Community-based group or day care<br>Social services and financial support<br>Do different providers work as part as one designated and integrated team or as part of different teams that work together?<br>What cross-sector, -setting or -profession activities take place to support integrated care?<br>Rounds or patient review meetings<br>Program operational or management meetings<br>Strategic planning and accountability

	range of settings with a range of organizations.	Budget setting an financial reporting<br>Joint Learning activities<br>How much are the work of the different team members co-located?<br>If team members are not co-located – what reinforces effective collaborative processes?<br><br>Prompt 2<br><br>What types of providers are involved in the program?<br>Nurses<br>Social Workers<br>Physician Assistants<br>Pharmacists<br>Behavioural supports through social work, psychiatry, or psychology<br>Secondary specialist care<br>Any other health human resources not yet described<br>What types of care settings and organizations are directly involved in the program?<br>Primary care in community<br>Inpatient acute medical care and surgical care<br>Home care<br>Nursing home<br>Supportive housing<br>Community-based group or day care<br>Social services and financial support<br>Do different providers work as part as one designated and integrated team or as part of different teams that work together?<br><br>What cross-sector, -setting or -profession activities take place to support integrated care?<br>Rounds or patient review meetings<br>Program operational or management meetings<br>Strategic planning and accountability<br>Budget setting an financial reporting<br>Joint Learning activities<br>How much are the work of the different team members co-located?

		If team members are not co-located – what reinforces effective collaborative processes?<br><br>Prompt 3<br><br>What types of providers are involved in the program?<br>Nurses<br>Social Workers<br>Physician Assistants<br>Pharmacists<br>Behavioural supports through social work, psychiatry, or psychology<br>Secondary specialist care<br>Any other health human resources not yet described<br>What types of care settings and organizations are directly involved in the program?<br>Primary care in community<br>Inpatient acute medical care and surgical care<br>Home care<br>Nursing home<br>Supportive housing<br>Community-based group or day care<br>Social services and financial support<br>Do different providers work as part as one designated and integrated team or as part of different teams that work together?<br><br>What cross-sector, -setting or -profession activities take place to support integrated care?<br>Rounds or patient review meetings<br>Program operational or management meetings<br>Strategic planning and accountability<br>Budget setting an financial reporting<br>Joint Learning activities<br>How much are the work of the different team members co-located?<br>If team members are not co-located – what reinforces effective collaborative processes?
	Care transitions between providers and settings is often	Prompt 1

	part of care for individuals with complex health and social care needs.<br><br>***#6 Prompt Question:*** Which **one** of the following statements do you think best describes the process for managing transitions in care in the program?<br><br>a. No structured protocols or coordinated process for care transitions across sectors or care settings<br>b. Protocol exists for some transitions but not others or protocols exist but are not routinely used<br>c. Clear protocol and strong commitment to ensuring smooth transitions across sectors or care settings	If there are no protocols, why have they not been developed?<br>What informally works?<br><br>Prompt 2<br><br>If there are protocols for specific transitions (e.g., at discharge from hospital, on admission to home care), what are the main features of these protocols?<br>Sharing patient care data<br>Drug reconciliation<br>Formal meetings of providers who span the transition<br>Patient and provider care engagement<br><br>If these are not used routinely, why are they not used?<br><br>Prompt 3<br><br>If there are protocols for specific transitions (e.g., at discharge from hospital, on admission to home care), what are the main features of these protocols?<br>Sharing patient care data<br>Drug reconciliation<br>Formal meetings of providers who span the transition<br>Patient and provider care engagement<br><br>If smooth care transitions are a major program commitment, why is this the case, what are the main features?<br><br>How is the effort coordinated and monitored?
	Sharing patient care and system performance data in a timely fashion is important.<br><br>***#7 Prompt Question:*** Which **one** of the following statements do you think best	Prompt 1<br><br>In the absence of clear processes or procedure, what “work arounds” are used to share data – e.g., text messaging or other methods to communicate?<br>Is there a plan to build an improved data sharing

	describes the processes and data infrastructure for timely data sharing in the program?<br><br>a. No clear process or procedure for sharing data across providers and organizations involved in patient care<br>b. Process and procedures exist to share information across providers and organizations involved in patient care, but not via shared access to single data infrastructure platform.<br>c. Partnering health care providers and organizations have timely access to shared data infrastructure platform.	capability?<br>Is data sharing a high priority for program sustainability and impact?<br><br>Prompt 2<br><br>Is there a documented protocol and set of procedures and policies for sharing important patient-care data across providers and settings in a timely fashion?<br>Is there operational common data platform for sharing data that allows for timely access to important patient-care data across providers though computers and/or hand held devices?<br><br>Prompt 3<br><br>Is there a documented protocol and set of procedures and policies for sharing important patient-care data across providers and settings in a timely fashion?<br>Is there operational common data platform for sharing data that allows for timely access to important patient-care data across providers though computers and/or hand held devices?<br>Is there a documented protocol and set of procedures and policies for sharing important system performance data across providers and settings in a timely fashion?
Patient engagement	Engagement of patients and caregivers can instrumental in the care of high needs populations. Engagement includes shared decision making, with active involvement of patients and caregivers with providers in developing the care plan, but also has a focus on self-management and support of caregivers. Patients and caregivers may also be involved in the co-design or the ongoing evaluation of the program.	
	#8 Prompt Question: Which **one** of the following statements do you think best describes the commitment to patient engagement in the	Prompt 1<br><br>If shared decision making is not a major component of the program, why is that?

	program?<br><br>a. Patient engagement not a clear component of organizational strategy, no clear processes to support shared decision-making in place<br>b. Patient engagement occurs to some degree, but no formal support for or training in shared decision-making processes.<br>c. Strong organizational support for, training in, and culture that promotes patient engagement	Prompt 2<br><br>Does the shared decision making include<br>Identification and recording patient defined goals for care<br>Development of individualized care planning based on input from patients and caregivers<br>Use of specific decision aids or shared decision making tools<br><br>Prompt 3<br><br>Does the shared decision making include<br>Identification and recording patient defined goals for care<br>Development of individualized care planning based on input from patients and caregivers<br>Use of specific decision aids or shared decision making tools<br><br>Does the commitment to training and creating a culture to support shared decision making, does it involve any of the following<br>A curriculum on shared decision making<br>A data infrastructure that supports shared decision making<br>Metrics or measurement tools that assess the extent to which there is a culture and process of shared decision making
	Patient self-management, self-efficacy and empowerment<br><br>***#9 Prompt Question:*** Which **one** of the following statements do you think best describes the process for supporting patient empowerment and self-	Prompt 1<br><br>If shared patient self-management is not a major component of the program, why is that?<br><br>Prompt 2<br><br>Commitment to patient self-management and empowerment includes

	management transitions in care in the program?<br><br>a. Promoting patient self-management and empowerment not a clear component of organizational strategy, no clear processes to support patient self-efficacy and empowerment in place<br>b. Promoting patient self-efficacy and empowerment occurs to some degree, but no formal support for or training in processes to promote patient self-efficacy and empowerment.<br>c. Strong organizational support for, training in, and culture that promotes patient self-efficacy and empowerment	Identification of specific conditions that are priorities for self-management<br>Inclusion of self-management into individualized care planning<br>Use of specific self-management tools<br><br>Prompt 3<br><br>Commitment to patient self-management and empowerment includes<br>Identification of specific conditions that are priorities for self-management<br>Inclusion of self-management into individualized care planning<br>Use of specific self-management tools<br><br>Commitment to training and creating a culture to support patient self-managements involves<br>A curriculum to train staff and patients on self-management<br>A data infrastructure that supports self-management<br>Metrics or measurement tools that assess the extent to which there is a culture and process of self-management
	Caregiver engagement, support and coaching<br>***#10 Prompt Question:*** Which **one** of the following statements do you think best describes the process for caregiver support in the program?<br><br>a. Caregiver support and coaching is not a clear component of organizational strategy, no clear processes to promote caregiver support and	Prompt 1<br><br>If caregiver support is not a major component of the program, why is that?<br><br>Prompt 2<br><br>The commitment to caregiver support and coaching includes<br>Identification of specific conditions that are priorities for caregiver support<br>Inclusion of caregiver support into individualized care planning<br>Use of specific caregiver support or coaching tools

	coaching in place<br>b. Caregiver support and coaching occurs to some degree, but no formal support for or training for caregiver support and coaching<br>c. Strong organizational support for, training in, and culture that promotes caregiver support and coaching	Prompt 3<br><br>The commitment to caregiver support and coaching includes<br>Identification of specific conditions that are priorities for caregiver support<br>Inclusion of caregiver support into individualized care planning<br>Use of specific caregiver support or coaching tools<br><br>The commitment to training and creating a culture to support self-managements involves<br>A curriculum to train staff on caregiver support<br>A data infrastructure that supports and coaches caregivers<br>Metrics or measurement tools that assess the extent to which there is caregiver burnout<br>Metris and measurement tools for assessing a culture and processes that support caregivers
Measures	We are interested in programs at different levels of maturity from programs that are in the pilot testing phase to programs that are very mature and that have been duplicated or replicated in many settings.<br><br>We understand that measures of success may vary with the maturity of the program and the approach to assessing the success of a program may vary depending on the perspective used in the assessment.	
History and state of maturity	In what year did this program admit its first client?<br><br>How many clients do you estimate have been admitted to this program since it was first started?<br><br>In the last 6 months how many new clients to you estimate have been admitted to this program?	
	Prompt Question: Which **one** of the statements do you think best describes the current state of this program?	Prompt 1<br><br>As a pilot program, what are the criteria, if any, for stopping the pilot or moving from pilot to an ongoing established program?

	a. Pilot program that is still evolving and not yet firmly established with ongoing funding<br>b. Established program with ongoing funding based at its initial site only<br>c. Established program with ongoing funding that has been replicated in sites	Does the funding for running the pilot program come from operational funds or research funds or both?<br><br>Prompt 2<br><br>Was this program established after being tried as a well-defined pilot or experimental program?<br>Did this program evolve from an existing established program without going through a pilot phase?<br>Was this program instituted as a new program and without a clear pilot or testing phase?<br><br>Prompt 3<br><br>How long was this initial program in place before it was duplicated in other sites or settings?<br>If this is a program that was expanded or duplicated, is the expansion for the same population or is it for a different patient population?
Measures of program success	We are interested in the current goals for the program. ***Prompt Question:*** Did the program goals include one or more of the following?<br><br>• Better health outcomes<br>• Better patient/caregiver experience<br>• Lower costs	• What were the specific goals for better health outcomes?<br>• What were the specific goals for better patient /caregiver experience?<br>• What were the specific goals for reduced costs?
	Many programs build in measures of program activities and the extent to which the program activities are consistent with the expectations. These measures can be used to assess program	Prompt 1<br><br>Why is there no routine collection of data on activities?<br>Is there no data on activities?<br>Is there data on activities but no operational interest in collecting these data routinely?

	implementation, in formative evaluations and in ongoing program monitoring<br><br>***Prompt Question:*** Which **one** of the following statements do you think best describes the extent to which the program routinely collects data program activities?<br><br>a. Data on program activities are rarely if ever collected in a routine fashion<br>b. Data on program activities have been used at times for assessing implementation or monitoring specific changes to the program<br>c. Data on program activities is routinely collected and used to monitor the program	Prompt 2<br><br>What specific program activity data have been collected?<br>Are those data used within the program for operational management?<br>Are those data used for external reporting?<br>Are those data available to us?<br><br>Prompt 3<br><br>What specific program activity data have been collected?<br>Are those data used within the program for operational management?<br>Are those data used for external reporting?<br>Are those data available to us?
	Some programs have undergone formal external evaluation, either as part of the requirements from the funder or as part of a research project.<br>***Prompt Question:*** Which **one** of the following statements do you think best describes the extent to which the program has been formally evaluated?<br><br>a. There has been no formal evaluation<br>b. There has been a formal evaluation by a funder<br>c. Here has been formal evaluation as part of a research study	Prompt 1<br><br>Why is there no formal evaluation?<br>Is this because formal evaluation is not normally done for this type of program?<br><br>Prompt 2<br><br>What metrics were used in the evaluation?<br>Did the metrics include health outcomes?<br>Did the metrics include patient or caregiver experience?<br>Did the metrics include costs?<br>What was he research design?<br>Did the design involve randomization to the program or a control group?<br>Did it involve comparison to a similar or matched setting or population?

		Did it involve before and after data?<br><br>Are the results of the evaluation available to us?<br><br>Prompt 3<br><br>What metrics were used in the evaluation?<br>Did the metrics include health outcomes?<br>Did the metrics include patient or caregiver experience?<br>Did the metrics include costs?<br>What was he research design?<br>Did the design involve randomization to the program or a control group?<br>Did it involve comparison to a similar or matched setting or population?<br>Did it involve before and after data?<br><br>Are the results of the evaluation available to us?

Part 2: Policy Environment - Instructions

The data collection tool is divided into two sections. The first focuses on policy innovation and the second on innovation in care delivery. Each section begins with a prompt question that identifies the types of innovation that are relevant to the program. Once you have selected all that are relevant, the tool focuses on collecting a bit more detail about the specific type of innovation.

Part 2: Policy environment		
Policy Innovation	Check all of the policy innovations to support integrated health and social care that you think make this program different from other programs that normally serve this population.<br><br>1. It involves a new way to finance health and social care by changing the way that funding for the program is provided or the way that the providers of care are paid<br>2. It creates a new staffing model for health and social care delivery or it redefines or creates new roles and responsibilities for staff<br>3. It creates a new governance structure or a new collaborative partnerships between health and social care organizations<br>4. It creates new ways for health and social care providers to collect or share data in a timely fashion	
	Finance	**Is there a well-defined budget or annual financial**

	(If yes to 1)	**statement for the program?**<br><br>• If so:<br>o How is that budget funded? Who pays for the program? How is that innovative or different from how health and social care programs are usually funded?<br>• If there is no clear budget or financial statement for the program:<br>Is it part of a larger program or initiative that does have a budget, financial statement and funding source? If so, how is the budget for the larger program financed? What makes the financing innovative?
		**Was there a budget for a pilot or initial set-up phase of the program?**<br><br>• If so, are the sources of funding the same source as the current funding?<br>If there was a shift from pilot funding to ongoing funding, who provided the initial funding and how was the program's level of ongoing funding determined?
		**How are the different members of program staff paid?**<br>• Are the members of program staff paid specifically for their involvement in the program?<br>• If physicians are paid for program activities, is that through their regular reimbursement mechanism or through a mechanism unique to this program?<br>• How are other members of staff paid?<br>What, if anything, makes staff reimbursement innovative?
	Staffing<br>(If yes to 2)	**Is there a clear staffing model or staffing plan for the program?**<br><br>• If so:<br>o What are the different staff categories and what is the proportional mix? Are staff full time or part time? If part time, what are their other commitments?<br>• If there is no clear formal staffing model:<br>o Who is involved in program delivery and how are they engaged in the program?

		How does the staffing for the program, in terms of professions or job descriptions, look different from other programs in the country?
		**How are the staff organized and prepared for the program?**<br><br>• How are the program organizational structure, policies or reporting lines innovative?<br>If members of staff are trained specifically to take part in this program, what is unique about that training?
	Governance structure (If yes to 3)	• **Is there a committee or a board that meets regularly and that brings together key stakeholders from the health and social care sectors to provide advice or oversight?**<br><br>o If yes, then what are its responsibilities and what is the membership? Is this structure unique to this program or is it common to other similar programs in your country?<br>If no, how do key health and social care partners work together and to whom is the program accountable? How is this process unique to this program?
		**Are data on program performance collected routinely and shared with funders, partners or governance bodies?**<br>• If so:<br>o What data are collected? Are these data unique to this program?<br>• Who receives and reviews the data?
	Information sharing (If yes to 4)	**What are the innovative policies or processes to collect data for patient care?**<br>• Are new types of data collected?<br>• If so, what are these new types of data?<br>How are these data collected? ?
		**What are the innovative policies or processes to share data for patient care in a more timely fashion?**<br>• Are there new policies that allow for data sharing?<br>Is there new data infrastructure that allows health and social care data to be shared in a more timely fashion? If so what is innovative in the data infrastructure?
Care Delivery Innovation	Check all of the care innovations that you think make this program different from other programs that normally serve this population.<br>1. It is different It terms of how much or who they pay for care	

	2. It is different in terms of who directly cares for them<br>3. It is different in terms of how involved they are in making care decisions<br>4. It is different in terms of data that they provide or how their data is shared by providers	
	If yes to 1	• Do patients and caregivers in this program see any difference in who pays for the health and social care that they receive compared to standard or usual care in your country?<br>• Do they end up paying more of less out of their own pockets?<br>• Do they see that there are payments being made for both health and social care?<br>• Do they understand who pays for health and social care?
	If yes to 2	• If so, what different care providers do they see?<br>Do they see one individual who acts as their care coordinator or navigator?
	If yes to 3	• If so, what types of decisions are they now involved in?<br>Are they likely to feel more accountable for their own care?
	If yes to 4	• What types of extra data do they provide?<br>How do they see data sharing as different?
