# Supplementary material for: Comparing International Models of Integrated Care: How Can We Learn Across Borders?
Source: Int J Integr Care. 2020 Apr 1;20(1):14. doi: 10.5334/ijic.5413 (PMC7147684; doi:10.5334/ijic.5413)
Supplement: Supplementary Material 2. — Integrated Care Case Study Descriptive Template – Modified Version. [file ijic-20-1-5413-s3.pdf]

Comparing international models of integrated care
RT1972, IJIC
Steele Gray et al 2020
Supplementary Materials

SUPPLEMENTARY MATERIAL 2

Integrated Care Case Study Descriptive Template – Modified Version
[New elements highlighted]

Introduction and guidelines

The following guide can be used to describe your model of integrated care in a way that is comparable to other cases. The framework has been used to describe dozens of cases across the globe, and has been validated through multiple research projects run out of academic institutions as well as the Commonwealth Fund in the United States. The template identifies core components of integrated care, separated into two parts with four sub-domains in each area. For each section you will note some definitions of concepts, but there is also an opportunity to define concepts based on local understandings and uses of terms. This template can be completed using one or multiple methods:

- Document review (memos, policies, network documents, white papers)
- Interviewing key stakeholders (providers, managers, patients and families)
- Secondary analysis of research (extracting data from other research and evaluation work)

Each sub-domain includes multiple choice questions followed by open ended questions to provide additional detail. We recommend using all data sources available to arrive at an answer to the question, and use the detailed descriptive sections to demonstrate why this is the most appropriate answer. Finally, we suggest that the description be reviewed by several individuals within the model at the front-line as well as at managerial levels to ensure key contextual factors are not missed.

Describe respondents and contributors

Upfront in your description provide an overview of:
1. The data sources used to complete the template (see above list, add other sources as applicable)
2. Who was engaged to help complete the template, including details such as (eg, what stakeholders were consulted):
    a. Role in the program (eg, provider, manager)
    b. Professional designation(s) (eg, nurse, social worker, physician) – if applicable
    c. Years working with the program
    d. What each person contribute (eg, filling out sections, reviewing/commenting, connecting to relevant individuals to provide information)

PART 1: PROGRAM DESIGN

Population of interest

Programs for high needs populations are often designed to identify specific populations that can be referred to the program.

Definition: Eligibility can be defined as how patients are considered as a candidate for inclusion into the model of program. [If another definition is more appropriate please elaborate].

Question 1: Which ***one*** *of the following statements do you think best describes the eligibility rules for the program?*

a. No firmly established rules exist, definition of eligible patients variable
b. Some rules in place to guide patient selection, but some flexibility remains in defining eligible patients
c. Clearly defined rules in place to define patient eligibility

If there are firmly established rules: Describe eligibility rules in terms of: How it is determined and defined, what drove those definitions (funding, policy), how they were developed, what is included (health and social care needs), are the rules flexible, who makes the decisions (providers, managers, patients and/or families), is there an appeal process, is there an audit process.

If there are no firmly established rules: Describe how eligibility is determined in the absence of clear rules, whether there is a plan to establish rules in the future, if not, why not.

In either case describe whether the model keeps track of how many get admitted and turned away, the reasons for refusal, what data infrastructures are in place to determine eligibility.

Normative integration prompts: Reflect on how eligibility criteria align, or conflict, with the values and aims of the programs, organization, and those delivering the programs.

Care Coordination

Definition - Care Coordination (from Wagner's Chronic Care Model; http://www.improvingchroniccare.org/index.php?p=The_Chronic_Care_Model&s=2): Deliberate organization of patient care activities between two or more participants involved in a patient's care to facilitate the appropriate delivery of health care services [If another definition is more appropriate please elaborate].

[This is the section that is bringing together referral and intake as part of our recommended changes to the template].

A common element of programs is single point or centralized process for referral, intake, or enrollment of patients in the program

Question 2: Program can use different strategies to apply referral, intake or enrollment. Which ***one*** *of the following statements do you think best describes the process for patient referral, intake and enrollment in this program?*

a. There is an informal process for referring and in-taking people into the program
b. Clear process exists but implemented variably by different program navigators or health care administrators
c. Single or established group of designated patient navigator(s) responsible for intake of all new patients in structured manner (eg, referral pathways built into data systems)

Definition - Care Navigator: A health professional that helps patients' access health and social care services available in the system. [If another definition is more appropriate please elaborate]

With regard to referring to the program, describe the referral process in terms of: how clients get referred (from others, self-referral, partners only, etc), how referrers learn about the program or model, tracking of referrals, whether there is variation in referral process, whether there is a data system to support the referral process.

With regard to in-taking once referred, describe how intake process in terms of: whether it is standardized (such as a single phone line), whether this is just part of "normal care" rather than a specialized process, which individual(s) conduct intakes and what are their roles in the organization (providers or administrator), are assessments and histories done as part of the process, how is intake data collected, stored and shared, is a "care navigator" part of this process and assigned at intake, when is intake available (eg, business hours) and how long does the process take, is the quality of intake monitored?

Normative integration prompts: Reflect on how referral and intake processes align, or conflict, with the values and aims of the programs, organization, and those delivering the programs.

Question 3: Many programs involve primary care providers (family physicians, general internists, geriatricians, pediatricians, nurse practitioners). Which one of the following statements do you think best describes the involvement of primary care providers in the program?

a. Limited involvement of primary care providers, including nurse practitioners.
b. Regular contact with at least one primary care provider, but primary care provider is not responsible for comprehensive management of patient needs through this program
c. Primary care provider clearly responsible for managing the care needs and care processes of the program's designated patients

Describe involvement of primary care in the program in terms of: who is "most responsible" for care and management of the patient, how primary care involvement has changed over time and future plans with regard to their role, how is primary care contact maintained, what roles primary care providers have (eg, drug reconciliation, planning transitions, reviewing care plans, monitoring patient outcomes, participating in care team meetings).

Normative integration prompts: Describe the relationship between primary care and other providers on the team.

Question 4: Programs often have a focus on integrating health and social care services. Which one of the following statements do you think best describes the integration of health and social care services in the program?

a. Programs involves a limited number of different health and social care provider types and is focused on specific social or health services or settings
b. Program involves multiple different health and social care provider types, care settings and organizations and provides both health and social care but not in highly coordinated manner
c. Program involves a wide range of health and social care provider types working in a coordinated system of delivery of integrated and comprehensive health and social care services in a range of settings with a range of organizations.

Describe the types of providers involved in the program in terms of: professional designation and roles (eg, nurses, social workers, physician assistants, pharmacists, behavioural supports, secondary specialists, volunteers), care settings involved in the programs (eg, primary care, in-patient acute medical and surgical care, home care, nursing home, supportive housing, community-based group or day care, social services, financial supports), do different providers work together as one team or do they work across multiple teams, what cross-sector activities take place (eg, rounds or patient review meetings, program operational or management meetings. strategic planning and accountability, budget setting and financial reporting, Joint Learning activities), are members co-located, what reinforces collaborative processes.

Normative integration prompts: Describe the relationship between the inter-disciplinary team (eg. Do teams work collaboratively, do they communicate well, are there strong relationships and trust).

Question 5: Care transitions between providers and settings is often part of care for individuals with complex health and social care needs. Which ***one*** *of the following statements do you think best describes the process for managing transitions in care in the program?*

Definition - Care Transition: Transfer of a patient between different care settings and health care providers during the course of an acute and chronic illness (definition from Health Quality Ontario, https://www.hqontario.ca/evidence-to-improve-care/quality-standards/view-all-quality-standards/transitions-between-hospital-and-home) [If another definition is more appropriate please elaborate].

a. No structured protocols or coordinated process for care transitions across sectors or care settings
b. Protocol exists for some transitions but not others or protocols exist but are not routinely used
c. Clear protocol and strong commitment to ensuring smooth transitions across sectors or care settings

If there are firmly established protocols: Describe if these protocols are for specific transitions eg, discharge from hospital), main features of these protocols (eg, sharing patient data, drug reconciliation, formal meetings, patient and provider engagement), and whether these are routinely used.

If there are no firmly established protocols: Describe why not and what informally works.
In either case describe whether smooth transitions are a major program commitment, and how the effort is coordinated and monitored.
Normative integration prompts: Reflect on how care transition protocols align, or conflict, with the values and aims of the programs, organization, and those delivering the programs.
Question 6: Sharing patient care and system performance data in a timely fashion is important. Which one of the following statements do you think best describes the processes and data infrastructure for timely data sharing in the program?

a. No clear process or procedure for sharing data across providers and organizations involved in patient care
b. Process and procedures exist to share information across providers and organizations involved in patient care, but not via shared access to single data infrastructure platform.
c. Partnering health care providers and organizations have timely access to shared data infrastructure platform.

If there are no clear processes and procedures: Describe how data is shared and any "work arounds" that are used and whether there is a plan to improve data sharing.
If there are clear processes and procedures, describe these processes in terms of: whether there is a documented protocol for sharing information, whether there are common data platforms available to support timely access to data.
In either case describe whether data sharing is a high priority for program sustainability and impact.
Patient and caregiver engagement
Engagement of patients and caregivers can instrumental in the care of high needs populations. Engagement includes shared decision making, with active involvement of patients and caregivers with providers in developing the care plan, but also has a focus on self-management and support of caregivers. Patients and caregivers may also be involved in the co-design or the ongoing evaluation of the program.
Definition – patient and caregiver engagement: "the process of building the capacity of patients, families, carers, as well as health care providers, to facilitate and support the active involvement of patients in their own care, in order to enhance safety, quality and people-centredness of health care service delivery." (definition from Patient Engagement: Technical Series on Safer Primary Care. Geneva: World Health Organization; 2016. Licence: CC BY-NC-SA 3.0 IGO). [If another definition is more appropriate please elaborate].
Question 8: Which one of the following statements do you think best describes the commitment to patient engagement, in particular shared decision-making processes, in the program?

a. Patient engagement not a clear component of organizational strategy, no clear processes to support shared decision-making in place
b. Patient engagement occurs to some degree, but no formal support for or training in shared decision-making processes.
c. Strong organizational support for, training in, and culture that promotes patient engagement

If shared decision-making is not a major component of the program: describe why not.

If shared decision-making is a major component of the program: Describe what activities shared decision-making includes (eg, identifying and recording patient goals, developing individualized care plans, use of specific decision aids or tools), whether there is training in shared decision-making (eg, a curriculum, a data infrastructure that supports shared decision making, metrics or measurement tools that assess the extent to which there is a culture and process of shared decision making), and whether training impacts the culture of supporting shared decision-making,

Question 9: Which ***one*** *of the following statements do you think best describes the process for supporting patient empowerment and self- management transitions in care in the program?*

a. Promoting patient self-management and empowerment not a clear component of organizational strategy, no clear processes to support patient self-efficacy and empowerment in place
b. Promoting patient self-efficacy and empowerment occurs to some degree, but no formal support for or training in processes to promote patient self-efficacy and empowerment.
c. Strong organizational support for, training in, and culture that promotes patient self-efficacy and empowerment

If patient self-management is not a major component of the program: describe why not.
If patient self-management is a major component of the program: Describe whether that commitment includes: identification of specific conditions that are priorities for self-management, inclusion of self-management into individualized care planning, use of specific self-management tools, training in patient self-management (eg, a curriculum, a data infrastructure that supports self-management, metrics or measurement tools that assess the extent to which there is a culture and process of self-management).

Question 10: Which one of the following statements do you think best describes the process for caregiver support in the program?

a. Caregiver support and coaching is not a clear component of organizational strategy, no clear processes to promote caregiver support and coaching in place
b. Caregiver support and coaching occurs to some degree, but no formal support for or training for caregiver support and coaching
c. Strong organizational support for, training in, and culture that promotes caregiver support and coaching

If caregiver support is not a major component of the program: describe why not.
If caregiver support is a major component of the program: Describe whether that commitment includes: identification of specific conditions that are priorities for caregiver support, inclusion of caregiver support into individualized care planning, use of specific caregiver support tools, training in caregiver support (eg, a curriculum to train staff on caregiver support, a data infrastructure that supports and coaches caregivers, metrics or measurement tools for assessing culture and process of caregiver support).

Measures
This section captures where programs are at in terms of their level of maturity from programs that are in the pilot testing phase to programs that are very mature and that have been duplicated or replicated in many settings. Measures of success may vary with the maturity of the program and the approach to assessing the success of a program may vary depending on the perspective used in the assessment.

Definition – program maturity: the state of a program in relation to an achievable goal state (definition from Groten L, Borgemans L, Vrijhoef, HJM. An Instrument to Measure Maturity of Integrated Care: A first validation study. *IJIC,* 18(1):10.

Describe the history and state of maturity in terms of:

- Year the program admitted its first client
- How many clients have been admitted since the program first started
- In the last 6 months how many new clients have been admitted to the program

Question 11: Which ***one*** *of the statements do you think best describes the current state of this program?*

a. Pilot program that is still evolving and not yet firmly established with ongoing funding
b. Established program with ongoing funding based at its initial site only
c. Established program with ongoing funding that has been replicated in sites

If it is a pilot program, describe the criteria for stopping the pilot or moving from pilot to an ongoing established program, and where funding for the pilot comes from (eg, operational funds, research funds, both, or other).

If this is an established program, describe whether it was established after a pilot or experimental program, and whether the program evolved since that pilot phase.

If this program has been replicated, describe how long it was in place prior to replication to new sites and the nature of that replication (eg, to a new geography, or different patient population).

Question 12: We are interested in the current goals for the program. Do the program goals include one or more of the following?

- Better health outcomes
- Better patient/caregiver
- Provider experience
- Lower costs

Describe the specific goals for better health outcomes, experience and reduced costs.

Normative integration prompts: Do performance measures captured align, or conflict, with the values and aims of the programs, organization, and those delivering the programs.

Measures of success: Many programs build in measures of program activities and the extent to which the program activities are consistent with the expectations. These measures can be used to assess program implementation, in formative evaluations and in ongoing program monitoring
Question 13: Which ***one*** *of the following statements do you think best describes the extent to which the program routinely collects data program activities?*

a. Data on program activities are rarely if ever collected in a routine fashion
b. Data on program activities have been used at times for assessing implementation or monitoring specific changes to the program
c. Data on program activities is routinely collected and used to monitor the program

If no data is collected about program activities, describe why not (eg, no routine collection, no available data, data is available on some aspects but not others).

If data is collected about program activities, describe what data on program activities is collected, how it is used (operational management, external reporting), and whether that data is routinely collected.

Question 14: Some programs have undergone formal external evaluation, either as part of the requirements from the funder or as part of a research project. Which ***one*** *of the following statements do you think best describes the extent to which the program has been formally evaluated?*

a. There has been no formal evaluation
b. There has been a formal evaluation by a funder
c. There has been formal evaluation as part of a research study

If there has been no formal evaluation, describe why (eg, is this not normally done for this type of program)?

If formal evaluations have been conducted, describe the metrics included (eg, health outcomes, experience, costs), the design of the evaluation (eg, research design using randomization, comparisons to similar groups or before and after data), and whether results are available.

Normative integration prompts: Do formal evaluations align, or conflict, with the values and aims of the programs, organization, and those delivering the programs.

PART 2: POLICY ENVIRONMENT

This part of the template is divided into two sections. The first focuses on policy innovation and the second on innovation in care delivery. Each section begins with a prompt question that identifies the types of innovation that are relevant to the program. Once you have selected all

that are relevant, the tool focuses on collecting a bit more detail about the specific type of innovation.

Policy Innovation

Check all of the policy innovations to support integrated health and social care that you think make this program different from other programs that normally serve this population. Identify in each instance what makes this innovative or different that status quo.

1. It involves a new way to finance health and social care by changing the way that funding for the program is provided or the way that the providers of care are paid

If there a well-defined budget or annual financial statement for the program, describe it in terms of: Who pays for the program? How is that innovative or different from how health and social care programs are usually funded?
If there is not clear budget of financial statement for the program, describe whether: it is part of a larger program or initiative that does have a budget, financial statement and funding source, how the budget for the larger program is financed, and what makes the financing innovative.
If there was a budget for a pilot or initial set up phase of the program, describe whether that funding relates to current funding, who provided initial funding versus ongoing funding, and how ongoing funding was determined.
Describe how program staff are paid (eg, based on their involvement in program, based on activities, through regular reimbursement mechanisms or new ones) and describe what, if anything, is innovative about this approach.
Normative integration prompts: Describe whether the funding model supports and is aligned to the overall vision of the model of care (eg, fee for service models may not well align with population health approaches).

2. It creates a new staffing model for health and social care delivery or it redefines or creates new roles and responsibilities for staff

If there is a clear staffing model or plan for the program, describe it in terms of: the different staff categories, the proportional mix, full-time and part-time status, and other commitments of providers
If there is not clear staffing model for the program, describe who is involved in program delivery and how they engaged in the program.
Describe how staffing for the program, in terms of professions or job descriptions, look different from other programs in the country?
Describe how staff are organized and prepared for the program in terms of: organizational structure, policies or reporting lines, training for staff (particularly how that training is unique).
Normative integration prompts: Describe whether the staffing model supports and is aligned to the overall vision of the model of care (eg, having inter-disciplinary teams to allow for whole person assessment and care delivery).

3. It creates a new governance structure or a new collaborative partnerships between health and social care organizations

If there is a committee or a board that meets regularly and that brings together key stakeholders from the health and social care sectors to provide advice or oversight, describe what are its

responsibilities and what is the membership, and whether this structure is unique to this program or is it common to other similar programs in your country.

If there is not a committee or a board, describe how do key health and social care partners work together and to whom is the program accountable, and how this process is unique to this program.

Describe what, if any, data on program performance is collected routinely and shared with funders, partners or governance bodies. Overview who receives and reviews this data and whether these data are unique to this program.

Normative integration prompts: Describe the values and aims of the committee or board, and how these were arrived at. Describe whether and how the work of the board or committee advance these values and aims for the model of care.

4. It creates new ways for health and social care providers to collect or share data in a timely fashion

Describe the innovative policies or processes to collect data for patient care in terms of: what new data are collected, and how these are collected.

Describe any innovative policies or processes to share data for patient care in a more timely fashion. These may include new data infrastructure that allows health and social care data to be shared in a more timely fashion, or other innovation information sharing methods or processes.

Care Delivery Innovation

Check all of the care innovations that you think make this program different from other programs that normally serve this population. Identify in each instance what makes this innovative or different that status quo.

1. It is different in terms of how much or who they pay for care

Describe it in terms of whether patients and caregivers in this program: see any difference in who pays for the health and social care that they receive compared to standard or usual care in your country; end up paying more or less out of pocket; see that there are payments being made for both health and social care; understand who pays for health and social care.

2. It is different in terms of who directly cares for them

Describe which care providers patients and caregivers see and whether there is one individual who acts as a care coordinator or navigator.

3. It is different in terms of how involved they are in making care decisions

Describe what types of decisions patients and families are involved in and whether this makes them more likely to feel accountable for their care.

4. It is different in terms of data that they provide or how their data is shared by providers

Describe what types of extra data are provided and how they view sharing data as different.
