# Supplementary material for: Comparing International Models of Integrated Care: How Can We Learn Across Borders?
Source: Int J Integr Care. 2020 Apr 1;20(1):14. doi: 10.5334/ijic.5413 (PMC7147684; doi:10.5334/ijic.5413)
Supplement: Supplementary Material 3. — Integrated Care Case Study Descriptive Template – Simplified Version. [file ijic-20-1-5413-s4.pdf]

SUPPLEMENTARY MATERIAL 3

Integrated Care Case Study Descriptive Template – Simplified Version

Introduction and guidelines

The following guide can be used to describe your model of integrated care in a way that is comparable to other cases. The framework has been used to describe dozens of cases across the globe, and has been validated through multiple research projects run out of academic institutions as well as the Commonwealth Fund in the United States. The template identifies core components of integrated care, separated into two parts with four sub-domains in each area, and asks primarily multiple choice questions regarding each domain. There is also space to provide additional detail through open ended questions.

This simplified version of the template is intended to be completed by a program manager with in-depth knowledge of the program. Those filling out the template may choose to consult with other members of their team for guidance as they like. There is a section at the end that allows you to identify who was involved in filling out the template.

For each section you will note some definitions of concepts, but there is also an opportunity to define concepts based on local understandings and uses of terms.

PART 1: PROGRAM DESIGN

Target population
Q1. Who is the target population for your program (check all that apply)

☐ Complex patients
☐ Older adults
☐ Kids
☐ Caregivers
☐ No specified target population

Q2. Identify what, if any, chronic conditions are targeted as part of your program (check all that apply)

☐ CHF
☐ Diabetes
☐ COPD
☐ Cognitive decline (dementia, Alzheimer's)
☐ Mental health
☐ Frailty
☐ No specified target condition

Eligibility
Definition: Eligibility can be defined as how patients are considered as a candidate for inclusion into the model of program.
If another definition is more appropriate please elaborate: [Open text]

*Q3. Which **one** of the following statements do you think best describes the eligibility rules for the program?*

☐ No firmly established rules exist, definition of eligible patients variable
☐ Some rules in place to guide patient selection, but some flexibility remains in defining eligible patients
☐ Clearly defined rules in place to define patient eligibility

Care Coordination
Definition – Care Coordination: Deliberate organization of patient care activities between two or more participants involved in a patient's care to facilitate the appropriate delivery of health care services.
If another definition is more appropriate please elaborate: [Open text]

Definition - Care Navigator: A health professional that helps patients' access health and social care services available in the system.

If another definition is more appropriate please elaborate: [open text]

Referral, intake, enrollment and assessment

Q4. Program can use different strategies to apply referral, intake or enrollment. Which one of the following statements do you think best describes the process for patient referral, intake and enrollment in this program?

☐ There is an informal process for referring and in-taking people into the program
☐ Clear process exists but implemented variably by different program navigators or health care administrators
☐ Single or established group of designated patient navigator(s) responsible for intake of all new patients in structured manner (eg, referral pathways built into data systems)

Q5. Which option best defines the entry process to your model of care

☐ Professional entry (eg, requires referral from a professional)
☐ Self-entry (eg, patients or families can self-refer into the program)
☐ Blended (eg, combination of both professional and self-entry)

Q6. Are assessments part of the intake process?

☐ Yes
☐ No
If yes, please list all assessment tools used: [open text]

Q7. Do assessments occur after the intake process?

☐ Yes, at regular intervals (eg, once every 3 months, at discharge)
☐ Yes, at random intervals (eg, when a provider decides it's time)
☐ No, assessments are only done at intake

Care teams

Q8. Many programs involve primary care providers (family physicians, general internists, geriatricians, pediatricians, nurse practitioners). Which one of the following statements do you think best describes the involvement of primary care providers in the program?

☐ Limited involvement of primary care providers, including nurse practitioners.
☐ Regular contact with at least one primary care provider, but primary care provider is not responsible for comprehensive management of patient needs through this program
☐ Primary care provider clearly responsible for managing the care needs and care processes of the program's designated patients

Q9. Programs often have a focus on integrating health and social care services. Which one of the following statements do you think best describes the integration of health and social care services in the program?

☐ Program involves a limited number of different health and social care provider types and is focused on specific social or health services or settings
☐ Program involves multiple different health and social care provider types, care settings and organizations and provides both health and social care but not in highly coordinated manner
☐ Program involves a wide range of health and social care provider types working in a coordinated system of delivery of integrated and comprehensive health and social care services in a range of settings with a range of organizations.

Q10. Identify the types of providers involved in your model (check all that apply)

☐ Primary care physicians/GPs/Family doctors
☐ Nurse practitioners
☐ Physician assistants
☐ Registered nurses
☐ Pharmacists
☐ Social workers
☐ Mental health workers (eg, psychologists, therapists)
☐ Behavioural supports
☐ Dietitians
☐ Chronic disease educators
☐ Rehabilitation (PTs, OTs, SLPs)
☐ Secondary specialists (eg, geriatricians, cardiologists)
☐ Personal support workers
☐ Volunteers

Q11. Identify the care settings involved in your program (check all that apply)

☐ Primary care
☐ In-patient acute medical and surgical care
☐ Home care
☐ Nursing home
☐ Supportive housing
☐ Community-based group or day care
☐ Palliative care
☐ Social services
☐ Financial supports

Q12. What cross sector activities take place (check all that apply)

☐ Rounds
☐ Patient review meetings
☐ Program operational or management meetings
☐ Strategic planning and accountability
☐ Budget setting
☐ Financial reporting

☐ Joint learning activities

Q13. Are providers in your model co-located?

☐ Yes, all
☐ Yes, some
☐ No

Care transitions
Definition - Care Transition: Transfer of a patient between different care settings and health care providers during the course of an acute and chronic illness.
If another definition is more appropriate please elaborate: [open text]

Q14. Care transitions between providers and settings is often part of care for individuals with complex health and social care needs. Which ***one*** *of the following statements do you think best describes the process for managing transitions in care in the program?*

☐ No structured protocols or coordinated process for care transitions across sectors or care settings
☐ Protocol exists for some transitions but not others or protocols exist but are not routinely used
☐ Clear protocol and strong commitment to ensuring smooth transitions across sectors or care settings

Sharing information
Q15. Sharing patient care and system performance data in a timely fashion is important. Which one of the following statements do you think best describes the processes and data infrastructure for timely data sharing in the program?

☐ No clear process or procedure for sharing data across providers and organizations involved in patient care
☐ Process and procedures exist to share information across providers and organizations involved in patient care, but not via shared access to single data infrastructure platform.
☐ Partnering health care providers and organizations have timely access to shared data infrastructure platform.

Patient and caregiver engagement
Definition – patient and caregiver engagement: "the process of building the capacity of patients, families, carers, as well as health care providers, to facilitate and support the active involvement of patients in their own care, in order to enhance safety, quality and people-centredness of health care service delivery."
[If another definition is more appropriate please elaborate: [open text]

Q16. Which one of the following statements do you think best describes the commitment to patient engagement, in particular shared decision-making processes, in the program?

☐ Patient engagement not a clear component of organizational strategy, no clear processes to support shared decision-making in place
☐ Patient engagement occurs to some degree, but no formal support for or training in shared decision-making processes.
☐ Strong organizational support for, training in, and culture that promotes patient engagement

Q17. What activities take place to support patient engagement (check all that apply)

☐ Goal-setting with patients
☐ Shared decision-making tools
☐ Developing individualized care plans
☐ Training for providers
☐ Training for patients
☐ Patient advisors on boards, or committees

Q18. Which ***one*** *of the following statements do you think best describes the process for supporting patient empowerment and self- management transitions in care in the program?*

☐ Promoting patient self-management and empowerment not a clear component of organizational strategy, no clear processes to support patient self-efficacy and empowerment in place
☐ Promoting patient self-efficacy and empowerment occurs to some degree, but no formal support for or training in processes to promote patient self-efficacy and empowerment.
☐ Strong organizational support for, training in, and culture that promotes patient self-efficacy and empowerment

Q17. What activities take place to support patient self-management (check all that apply)

☐ Educational materials and information sharing
☐ Chronic disease management support groups
☐ Self-monitoring supports (eg, blood glucose monitors, mobile apps)
☐ Self-management part of care planning
☐ Training for patients
☐ Training for providers

Q18. Which one of the following statements do you think best describes the process for caregiver support in the program?

☐ Caregiver support and coaching is not a clear component of organizational strategy, no clear processes to promote caregiver support and coaching in place
☐ Caregiver support and coaching occurs to some degree, but no formal support for or training for caregiver support and coaching
☐ Strong organizational support for, training in, and culture that promotes caregiver support and coaching

Q19. What activities take place to support patient engagement (check all that apply)

☐ Caregiver support built into care plans
☐ Use of caregiver support tools
☐ Information, advice or guidance provided to caregivers
☐ Training for caregivers
☐ Training for patients
☐ Respite programs
☐ Caregiver advisors on boards, or committees

Program maturity

Definition – program maturity: the state of a program in relation to an achievable goal state.
[If another definition is more appropriate please elaborate: [open text]

*Q20. Which **one** of the statements do you think best describes the current state of this program?*

☐ Pilot program that is still evolving and not yet firmly established with ongoing funding
☐ Established program with ongoing funding based at its initial site only
☐ Established program with ongoing funding that has been replicated in sites

OR

Q20. What is the maturity level of your program?

☐ Initial/start up phase (eg, planning phase)
☐ Experimental and execution phase (eg, pilot phase)
☐ Expansion and monitoring phase (eg, expanding to new sites)
☐ Consolidation and transformation phase (eg, established sustained program)

Program measures

Q21. We are interested in the current goals for the program. Do the program goals include one or more of the following?(check all that apply)

☐ Better health outcomes
☐ Better patient/caregiver experience
☐ Better provider experience
☐ Lower costs

*Q22. Which **one** of the following statements do you think best describes the extent to which the program routinely collects data program activities?*

☐ Data on program activities are rarely if ever collected in a routine fashion
☐ Data on program activities have been used at times for assessing implementation or monitoring specific changes to the program
☐ Data on program activities is routinely collected and used to monitor the program

Q23. If data is collected, how is it used?(check all that apply)

☐ Data on program activities is used to evaluate success
☐ Data on program activities is used to demonstrate accountability to stakeholders
☐ Data on program activities is used to drive funding decisions
☐ Data on program activities is used to drive program decisions

Q24. Some programs have undergone formal external evaluation, either as part of the requirements from the funder or as part of a research project. Which ***one*** *of the following statements do you think best describes the extent to which the program has been formally evaluated?*

☐ There has been no formal evaluation
☐ There has been a formal evaluation by a funder
☐ There has been formal evaluation as part of a research study

PART 2: POLICY ENVIRONMENT

Policy Innovation

Q24. What best describes the funding model for the program?

☐ There is a well-defined budget or annual financial statement for the program
☐ The budget for the program is part of a larger program that does have a budget or funding source
☐ There was a budget for a pilot or initial set up of the program, but no ongoing funding

Q25. Where does, or did, funding for the program come from?

☐ Single source
☐ Multiple sources

Q26. Who does, or did, fund the program? (check all that apply)

☐ Government/ministry
☐ Regional board
☐ Private health insurers
☐ Not-for-profit agency
☐ Client fees
☐ Other: [open text]

Q27. What best describes the staffing model for the program?

☐ Staff employed by the program
☐ Staff employed by their parent organizations

Q28. What best describes the governance model for the program?

☐ Shared governance structure – all partners involved
☐ Partly shared governance structure – some partners involved
☐ Single organization governs the program

Q29. Describe the level of innovativeness, or how different your program is as compared to the status quo of care delivery in your region:

	Very different	Somewhat different	Not at all different	Don’t know
Who pays for care				
Who cares for patients				
Who is involved in care decisions				
How data is shared				

<u>DESCRIBE RESPONDENTS</u>

1. Who was engaged to help complete the template, including details such as:
    a. Role in the program (eg, provider, manager)
    b. Professional designation(s) (eg, nurse, social worker, physician) – if applicable
    c. Years working with the program
    d. What each person contribute (eg, filling out sections, reviewing/commenting, connecting to relevant individuals to provide information)
